# Palmitoylated COX-2^Cys555^ reprogrammed mitochondrial metabolism in pyroptotic inflammatory injury in patients with post-acute COVID-19 syndrome

**DOI:** 10.1016/j.jare.2025.05.005

**Published:** 2025-05-09

**Authors:** Jia-Shen Wu, Chi-Yu Xu, Su-Min Mo, Xin-Mou Wu, Ze-Bang Du, Lin Che, Yi-Ling Zhang, Kai-Li Yang, Ting-Dong Li, Sheng-Xiang Ge, Tian-Ying Zhang, Zhong-Ning Lin, Yu-Chun Lin

**Affiliations:** State Key Laboratory of Vaccines for Infectious Diseases, Xiang An Biomedicine Laboratory, Xiang’an Hospital of Xiamen University, National Innovation Platform for Industry-Education Integration in Vaccine Research, School of Public Health, Xiamen University, Xiamen 361102, China

**Keywords:** SARS-CoV-2 spike protein, COX-2 palmitoylation, Mitochondrial metabolic reprogramming, NLRP3 inflammasome activation, Lung inflammatory injury, Post-acute COVID-19 syndrome (PACS)

## Abstract

**Introduction:**

The complex interplay between protein palmitoylation, mitochondrial dynamics, and inflammatory responses plays a pivotal role in respiratory diseases. One significant feature of post-acute coronavirus disease 2019 (COVID-19) syndrome (PACS) is the occurrence of a storm of inflammatory cytokines related to the NOD-like receptor protein 3 (NLRP3). However, the specific mechanisms via which palmitoylation affects mitochondrial function and its impact on the NLRP3 inflammasome under pathological respiratory conditions remain to be elucidated.

**Objective:**

This study aimed to investigate how protein palmitoylation influences inflammatory responses and mitochondrial dynamics in respiratory diseases, such as those induced by the SARS-CoV-2 spike S protein in PACS, thereby providing a therapeutic target for inflammatory lung injury.

**Methods:**

*In vivo* experiments were conducted using AdV5-pADM-CMV-COVID-19-S (AdV5-S) nasal drip-treated C57BL/6 mice to assess NLRP3 inflammasome activation and inflammatory response. *In vitro* experiments were performed using pCMV-S-transfected human lung epithelial BEAS-2B cells to analyze the effects of DHHC5-mediated palmitoylation of cyclooxygenase-2 (COX-2) at cysteine 555 (COX-2^Cys555^) on mitochondrial metabolism and NLRP3 inflammasome activation.

**Results:**

Palmitoylation of COX-2^Cys555^ enhanced its interaction with hexokinase 2 (HK2) to regulate mitochondrial metabolic reprogramming, leading to NLRP3 inflammasome activation and pyroptosis. Pharmacological and genetic suppression of palmitoylation diminished the mitochondrial localization of palmitoylated COX-2 and its interaction with HK2, thereby reducing mitochondrial metabolic reprogramming. Furthermore, genetic intervention targeting DHHC5 (sh*Dhhc5*) alleviated NLRP3 activation and pyroptosis, mitigating the chronic inflammatory damage associated with PACS.

**Conclusion:**

This study highlights the regulatory role of COX-2^Cys555^ palmitoylation in mitochondrial metabolism and lung inflammatory injury, and suggests potential therapeutic targets to combat respiratory pathogenesis linked to palmitoylated COX-2.

## Highlights

• NLRP3-dependent pyroptosis promotes lung injury via SARS-CoV-2 S protein expression.
• Palmitoylated COX-2^Cys555^ interacts with HK2 to facilitate reprogramming of mitochondrial metabolism.
• DHHC5-mediated palmitoylation of COX-2/HK2 activates NLRP3-dependent pyroptosis.
• Genetic intervention targeting DHHC5 represents a potential approach for long-term treatment of COVID-19.

## Graphical abstract

**Figure:**
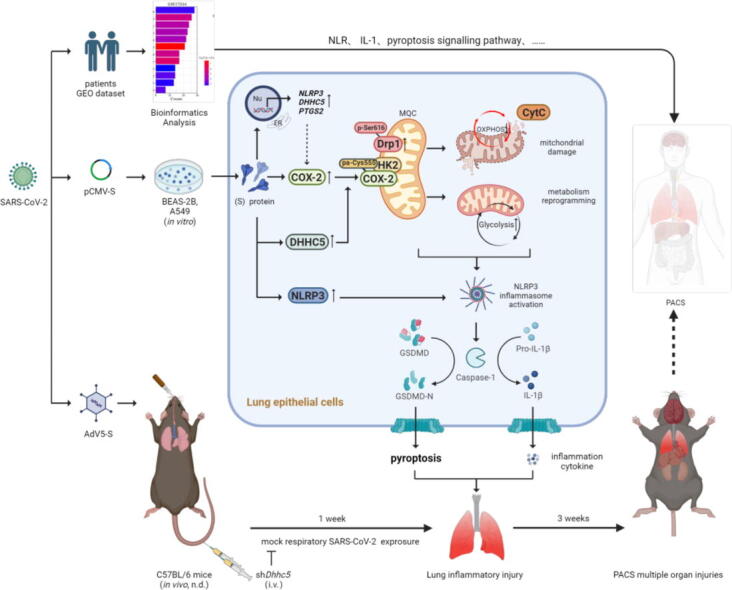

## Introduction

Protein lipidation, particularly palmitoylation, in which palmitoyl groups are covalently attached via thioester bonds to cysteine residues, is a critical post-translational modification that influences protein properties. This process is regulated by palmitoyl S-acyltransferases (PATs) from the DHHC protein family and plays an essential role in various diseases, including inflammatory diseases, infectious diseases, and cancer [[1], [2], [3]]. Enveloped viruses often exhibit S-acylation of their glycoproteins, and evidence indicates that palmitoylation is critical in viral infections. This is particularly notable due to the presence of conserved cysteine motifs in the transmembrane region of the coronavirus spike (S) protein [4]. In particular, palmitoylation of the severe acute respiratory syndrome coronavirus 2 (SARS-CoV-2) S protein is essential for viral fusion with host cells. It also facilitates the trafficking of viral glycoproteins, thereby promoting the assembly and budding of progeny virions from infected epithelial cells [[5], [6], [7], [8]]. Moreover, palmitoylation is vital for targeting proteins to lipid rafts, which act as membrane platforms for viral entry, assembly, and budding [9,10]. Inhibition of specific protein palmitoylation has emerged as a promising strategy for treating viral diseases, with the zDHHC protein family and PATs emerging as potential drug targets [11]. However, the function of protein palmitoylation in the regulation of lung injury remains unclear.

Cyclooxygenases (COXs), including cyclooxygenase-1 (COX-1) and cyclooxygenase-2 (COX-2), are rate-limiting enzymes that catalyze the conversion of arachidonic acid to prostaglandins [12]. COX-2 plays a pivotal role in regulating inflammation and immune responses, including pain, fever, and cancer [13,14]. The expression of COX-2 is tightly regulated by nuclear factor-kappa B (NF-κB), MAPK pathways, and other signaling cascades, which activate downstream enzymes such as iNOS and VCAM-1, contributing to airway inflammation in respiratory diseases [15,16]. Our previous studies have highlighted the critical role of COX-2 in mitochondrial quality control and function, demonstrating that dephosphorylation of COX-2 at serine 601 and its subsequent mitochondrial transport initiate pyroptosis in hepatocytes, underscoring the importance of post-translational modifications of COX-2 in regulating mitochondria-associated inflammatory injury [17]. Mitochondrial COX-2 influences mitochondrial dynamics through dynamin-related protein 1 (Drp1) and is associated with proteins involved in mitochondrial function and metabolism [18,19]. Emerging evidence indicates that SARS-CoV-2 infection triggers metabolic reprogramming in lung epithelial cells, contributing to chronic inflammation by upregulating pyruvate kinase M2 (PKM2) expression, which is linked to increased glycolysis and the accumulation of glycolysis intermediates [20,21]. Notably, the SARS-CoV-2 S protein elevates COX-2 expression in lung epithelial cells, initiating and exacerbating the inflammatory characteristics of coronavirus disease 19 (COVID-19) [21,22]. These results indicate that targeting of mitochondrial COX-2 may offer a novel therapeutic strategy against lung inflammatory injury. However, whether this is related to regulation of palmitoylation remains unclear.

While the immediate symptoms of COVID-19 have garnered substantial attention, the long-term health consequences, termed long COVID or post-acute COVID-19 syndrome (PACS), are increasingly being recognized. These prolonged effects encompass a range of symptoms, including respiratory difficulties, cardiovascular complications, neurological disorders, and chronic fatigue, highlighting the complex nature of COVID-19 [[23], [24], [25]]. Inflammatory injuries, such as pyroptosis induced by NOD-like receptor protein 3 (NLRP3) inflammasome activation, have been reported during both acute infection and PACS [19]. Dysregulation of palmitoylation, which is essential for inflammatory responses and mitochondrial function, may contribute to the pathogenesis of long COVID. Recent findings identifying zDHHCs as mediators of S-acylation in viral proteins of influenza and SARS-CoV-2 suggest that targeting of these enzymes may provide antiviral strategies [26]. Studies have indicated that the palmitoylation/depalmitoylation cycle is vital for the progression and treatment of lung diseases, including COVID-19 [27]. Modulation of COX-2 activity, potentially through palmitoylation, may provide effective therapeutic strategies for inhibiting viral replication and reducing inflammatory damage. However, the role of palmitoylation induced by the SARS-CoV-2 S protein in the mitochondrial transport of COX-2 remains unknown. By focusing on the regulatory mechanisms of protein palmitoylation, particularly of COX-2, therapies can be developed to more effectively alleviate lung inflammatory injury, addressing both acute infections and chronic inflammation.

This study aimed to elucidate the interplay between palmitoylated COX-2, mitochondrial metabolic reprogramming, activation of the NLRP3 inflammasome, and the resulting inflammatory lung injury induced by the SARS-CoV-2 S protein, using both *in vitro* and *in vivo* methods. We aimed to provide valuable insights into the pathogenesis of acute lung inflammatory injury and highlight the importance of neurological manifestations in post-acute conditions. Considering the clinical implications of persistent symptoms, we believe that our findings will contribute to the development of targeted strategies for the prevention and treatment of chronic lung inflammatory injury.

## Materials and methods

### Bioinformatics analysis

The Gene Expression Omnibus (GEO) databases (GSE172114, GSE147507, and GSE153277) were used to investigate pathway enrichment (https://www.ncbi.nlm.nih.gov/geo/). The GSE172114 dataset contained whole blood RNA sequencing data from 47 patients with severe COVID-19 who progressed to acute respiratory distress syndrome and 22 healthy individuals. The GSE147507 dataset contained RNA sequencing data from SARS-CoV-2-infected human lung epithelial cells (A549 cells). The GSE153277 dataset was derived from RNA sequencing data of SARS-CoV-2-infected type II alveolar epithelial cells differentiated from human pluripotent stem cells, with samples collected at day 1 (1 d.p.i.) and day 4 (4 d.p.i.) post-infection from both the SARS-CoV-2 infection group (n = 6) and the uninfected control group (n = 3). Quality control, normalization, and data pre-processing were performed as required. The log2 (fold change) and *P*-value thresholds were set at 1.0 and 0.05, respectively. Functional enrichment analyses, including Gene Ontology (GO) analysis and pathway analyses, were performed to elucidate the biological processes and pathways associated with the identified differentially expressed genes (DEGs). DEGs were screened to provide clues for subsequent experiments. Raw histological data were normalized using an online bioinformatics website (https://hiplot.com.cn/) and visualized as bubble and violin plots. A correlation network diagram was used to identify the connections among the DEGs.

### Animals and establishment of models

C57BL/6 mice (6 weeks old) were obtained from the SLAC Laboratory Animal Co., Ltd. (Shanghai, China). C57BL/6 mice (both male and female, weighing 20 − 25 g) were used for the construction of the acute lung injury (ALI) model and for the infection with AdV5-pADM-CMV-COVID-19-S (AdV5-S) (Weizhen, Jinan, Shandong, China). The mice were housed in groups of five per cage, maintained on a 12-hour light/dark cycle, and provided with ad libitum access to food and water. All animal experiments were conducted in accordance with the guidelines of Xiamen University Animal Ethical Committee. The animal experiment protocols were approved by the Ethical Committee for Animal Experimentation at Xiamen University (Ethical Approval Number: XMULAC20200201, dated 2020–02-01).

The ALI model was induced by intratracheal injection of lipopolysaccharides (LPS, 10 mg/kg), while the control group received 50 μL of sterile normal saline (NS). After 24 h of LPS or NS treatment, the mice were infected in the same manner with AdV5-S (1 × 10^7^ PFU) or AdV5-empty vector [28].

For the gene knockdown experiments, intravenous tail injections of sh-*NC* (NC) or sh*Dhhc5* were performed. The shRNA constructs were collected, encapsulated in DOTAP:Chol liposomes, and administered via tail vein injection. The plasmid harboring sh*Dhhc5* was extracted, and its concentration was determined using an endotoxin-free plasmid extraction kit, yielding a stock solution of 2000 ng/μL. DOTAP lipids were dissolved in 1 mL of ethanol to create a 20 mM stock solution, which was mixed using a probe sonicator at 40 % amplitude for 2 min (10 s pulses with 5 s intervals) before use. The lipid/sh*Dhhc5* mixture was prepared by combining 20 μL of the lipid stock, 25 μL of sh*Dhhc5* stock, and 55 μL of a 5 % glucose solution in a total volume of 100 μL. The mouse tail was secured using a visual mouse tail intravenous injection device, and 100 μL of the shRNA suspension was injected intravenously. Each group consisted of six mice. Lung tissues were collected for histological and gene expression analyses after 7 days. The target sequences of sh*Dhhc5* are shown in Table S1.

### Assessment of olfactory function in mice expressing SARS-CoV-2 S protein

The food discovery test was used to evaluate the olfactory function in mice. For 5 days prior to the experiment, mice were provided with cookies for 1 h daily to familiarize them with the food. Food was restricted for 12 h prior to testing. On the testing day, mice were placed in a clean cage and acclimatized for 20 min. The cookies were then placed in one corner of the cage, and the mice were positioned in the opposite corner. The time taken by the mice to locate the food was recorded.

### Bronchoalveolar lavage fluid (BALF) assay

Mice were euthanized using an isoflurane overdose and subsequently disinfected with 75 % ethanol. An incision was made near the trachea, and a scalp needle was used to puncture the middle part of the tracheal cartilage rings, and a catheter was then inserted into the trachea. Sterile physiological saline was slowly injected into the lungs, followed by simultaneous aspiration of the solution. To further separate the cellular and non-cellular components, the BALF samples were centrifuged at 4 °C and 400 × *g* for 10 min. The retrieved lavage fluid was stored on ice until further analysis.

### Histopathology and immunohistochemistry (IHC) analysis

Tissue specimens were fixed with 4 % paraformaldehyde and embedded in paraffin for thin sectioning. The paraffin-embedded tissue sections were subjected to dewaxing using xylene and rehydrated through graded ethanol solutions. For histology, tissue sections were stained with hematoxylin and eosin (H&E) to visualize cellular morphology and tissue structure. Masson's staining was performed to assess collagen content and tissue fibrosis. To detect the indicated proteins in the tissue sections, IHC staining was conducted as previously described [19]. In brief, sections were dewaxed, rehydrated, and antigen retrieval was performed, followed by the use of the UltraSensitive™ SP (Mouse/Rabbit) IHC Kit (Maixin Biotech, Fuzhou, Fujian, China), according to the manufacturer's instructions. Sections were incubated with the indicated primary antibodies, including anti-COX-2, anti-p-Drp1^Ser616^, anti-Mfn2, and anti-IBA1 antibodies, at 4 °C overnight. Visualization of protein expression and distribution was achieved using a 3,3′-diaminobenzidine (DAB) stain. Images were captured using a TS100 inverted microscope (Nikon, Tokyo, Japan). The detailed information on the primary antibodies is listed in Table S2.

### Cell culture and DHHC5 knockdown

Immortalized human lung cell lines, A549 and BEAS-2B, were preserved and maintained in our laboratory. Both cell lines were cultured in Dulbecco's Modified Eagle's medium (DMEM; Gibco, New York, NY, USA) supplemented with 10 % fetal bovine serum (FBS; Gibco, New York, NY, USA) and 1 % penicillin–streptomycin (Gibco, New York, NY, USA). All cell lines were maintained at 37 °C in a 5 % CO_2_ humidified incubator (Thermo Fisher, Waltham, MA, USA). Stable COX-2-knockdown cell lines were constructed as described previously [19]. Briefly, a pLKO.1 lentiviral short hairpin RNA (shRNA) vector targeting *PTGS2* (COX-2) was used to establish stable COX-2-knockdown cell lines. The *PTGS2*-shRNA targeting sequence (5′-CCAGGGCTCAAACATGATGTT-3′) corresponding to positions 665–685 of the human *PTGS2* mRNA was designed using the RNAi Consortium website. The pLKO.1-*PTGS2*-shRNA recombinant plasmid was used to construct stably expressing cells via lentiviral infection and puromycin selection, while the pLKO.1 vector was used as a control. To establish stable DHHC5-knockdown cell lines, small guide RNA (sgRNA)-coding cDNAs targeting *DHHC5* were designed and synthesized for the construction of Lenti-Cas9-*DHHC5* recombinant plasmids. The *DHHC5-*sgRNAs were cloned into the LentiCRISPRv2 vector. Lenti-Cas9-*DHHC5* expressing constructs or the negative control (NC) plasmid, Lenti-Cas9-NC, were transfected into HEK-293T cells using the Lipofectamine 2000 reagent, according to the manufacturer's instructions. The transfected cells were selected using 0.6 μg/mL puromycin to generate a polyclone of cells with stable knockdown of DHHC5, termed A549-Cas9-*DHHC5* cells; A549-Cas9-NC cells were used as controls. The target sequences of *DHHC5*-sgRNA-1 and *DHHC5*-sgRNA-2 are listed in Table S1.

### Overexpression of COX-2 with wild type or mutants

All mutants of potential palmitoylated COX-2 sites (C9S, C54S, C145S, C526S, C555S) were generated by site-directed mutagenesis using the KOD-Plus Mutagenesis kit (Toyobo, Osaka, Japan), following the manufacturer's instructions. Construction of stable wild type (WT) or mutant (MUT) COX-2-overexpressing cells was carried out as previously described [18]. Briefly, the *PTGS2* coding sequence was amplified from a cDNA library (a kind gift from Dr. Jia-Huai Han, Xiamen University, China) using RT-PCR with specific primers (FP: 5′-ATTAGGATCCATGCTCGCCCGCGCCCT-3′ and RP: 5′-GAGTCGACTTACTTATCGTCGTCATCCTTGTAATCCAGTTCAGTCGAACGTT-3′). The fragment was then cloned into the pLVX-Puro lentiviral vector. Stable COX-2 (WT) cells (A549-*PTGS2* and BEAS-2B-*PTGS2*) were established via lentiviral infection and puromycin selection. The same method was used to construct stable expression cells for each mutant plasmid.

### Lactate dehydrogenase (LDH) release and cytokine quantification in culture supernatants

To evaluate the effect of SARS-CoV-2 S protein expression on cell membrane integrity, LDH release was measured. Briefly, cells were seeded in a 96-well plate and treated as indicated. 120 μL of culture supernatant was collected and transferred to a new 96-well plate. LDH detection was performed according to the manufacturer's protocol (Beyotime, Shanghai, China). Absorbance was measured at 490 nm using a multifunctional ELISA reader (CLARIOstar, Ortenberg, Germany), with background correction at 600 nm.

The concentrations IL-1β and IL-6 were measured using ELISA kits (Abclonal, Wuhan, Hubei, China), according to the manufacturer's protocols.

### Cell viability

Cell viability was assessed using the MTT assay, following established protocols [29]. Briefly, after the respective treatments, absorbance was measured using a microplate spectrophotometer system (BMG LabTech, Ortenberg, Germany) at a wavelength of 490 nm. The cell viability rate (%) was calculated.

### RNA isolation and quantitative real-time PCR (qRT-PCR)

Total RNA was extracted from cells using TRIzol reagent (TaKaRa, Otsu, Japan). cDNA was synthesized from 1 μg of total RNA using the PrimeScript^TM^ RT reagent kit (TaKaRa, Otsu, Japan). qRT-PCR was performed as described in our previous study [19], using the primers listed in Table S1. The 2^-ΔΔCt^ method was employed to determine the relative mRNA levels of target genes. The primer information is listed in Table S1.

### Western blotting (WB)

WB analysis was performed as described previously [19]. Briefly, SDS-PAGE gels were prepared according to standard laboratory protocols. Proteins were separated by gel electrophoresis based on their molecular weights. Following electrophoresis, proteins were transferred to a PVDF membrane (Millipore, MA, USA). The membrane was blocked with a 5 % skimmed milk solution, followed by incubation with primary antibodies specific to the target proteins. This step was followed by incubation with secondary antibodies conjugated to enzymes. Luminescent signals were developed on the membrane to visualize and quantify the presence of the indicated proteins. Detailed information on the primary antibodies, including manufacturers, catalog numbers, dilutions used in WB, molecule weights, and species, is listed in Table S2.

### Immunofluorescence (IF) assay

Cells mounted on coverslips were stained with MitoTracker Red (Thermo, Waltham, MA, USA), LysoTracker Red (Thermo, Waltham, MA, USA), or Dio to label cellular components or structures. Following staining, the cells were incubated with primary antibodies, including anti-GSDMD and anti-COX-2 antibodies, at a dilution of 1:200. A high-sensitivity laser confocal microscope (Zeiss, Jena, Germany) was employed for imaging and analysis of the colocalization of GSDMD with cell membrane, as well as the expression and colocalization of COX-2, as described in a previous study [19]. Detailed information on the primary antibodies is listed in Table S2.

### Acyl-resin-assisted capture (Acyl-RAC) assay

The Acyl-RAC assay was used to detect the levels of palmitoylated COX-2, according to a previously described protocol with minor modifications [30,31]. Cells were lysed in 400 μL of lysis buffer (0.5 % Triton X-100, 25 mM HEPES, 25 mM NaCl, 1  mM EDTA, pH 7.4, and a protease inhibitor cocktail) and incubated for 30 min at room temperature. Then, 200 μL of blocking buffer (100 mM HEPES, 1 mM EDTA, 87.5 mM sodium dodecyl sulfate (SDS), and 1.5 % [v/v] S-methyl methanethiosulfonate) was added and vortexed for 4 h at 40 °C. Proteins were precipitated with acetone and resuspended in a buffer (100 mM HEPES, 1 mM EDTA, 35 mM SDS). The resuspended proteins were then incubated with a final concentration of 400 mM hydroxylamine (HAM) in pre-activated beads for overnight protein capture. As a negative control, Tris was used in place of HAM. After overnight incubation on a rotating wheel and washing, the proteins were eluted with 40 μL SDS sample buffer containing β-mercaptoethanol at 95 °C for 5 min. Protein samples were separated using SDS-polyacrylamide gel electrophoresis and analyzed using WB. A fraction of the cell lysate (total cell extract) was used as an input for comparison. To validate the COX-2 palmitoylation induced in cells, 2-BP, a non-specific palmitoylation inhibitor, was used. Additionally, ML348, purchased from Yuanye (Shanghai, China), was used as a depalmitoylation inhibitor.

### Isolation of mitochondrial and cytoplasmic fractions and mitochondrial protein immunoprecipitation (Mito-IP)

Mitochondrial and cytoplasmic fractions were separated using a mitochondrial isolation kit (Enzo Life, New York, NY, USA) according to the manufacturer's instructions, as previously described [19]. The entire process was conducted on ice, and unless otherwise specified, the centrifugation temperature was maintained at 4 ℃. Briefly, at least 5 × 10^7^ cells per group were harvested, washed with cold PBS, and centrifuged at 600 × g for 5 min to remove debris. The cell pellet was resuspended in mitochondria isolation buffer, incubated on ice for 10 min, and then homogenized. The lysate was centrifuged at 600 × g for 10 min, and the supernatant was further centrifuged at 12,000 × g for 10 min to isolate mitochondria in the pellet and the cytoplasmic fraction in the supernatant. The mitochondrial pellet was resuspended in lysis buffer and centrifuged again at 12,000 × g for 10 min for purity assessment. Protein concentrations in both fractions were measured using the BCA assay. To detect the levels of COX-2 and hexokinase 2 (HK2) proteins in both mitochondrial and cytoplasmic fractions, WB analysis was performed. COX-IV and GAPDH were used as loading controls, respectively. Mito-IP was conducted according to established protocols. Briefly, SureBeads (100 μL) (Bio-Rad, Hercules, CA, USA) were washed three times with 1 × PBST (PBS plus 0.1 % Tween 20), and the supernatant was removed using a magnetic separator. Then, 200 μL of primary antibodies (anti-COX-2 and anti-Flag) were added to the SureBeads and incubated on a rotary rocking bed for 20 min. Mitochondrial fractions were applied to the beads at room temperature for 1 h. The beads were washed three times with 1 × PBST, and the proteins were eluted using 1 × SDS buffer for subsequent WB analysis.

### Transmission electron microscopy (TEM) assay

Cells were collected and centrifuged at 1000 × *g* for 5 min to form a pellet. The samples were then fixed by incubation in a 1.5 % osmium tetroxide solution for 2 h. Following preparation into ultrathin sections, the sections were loaded onto a transmission electron microscope (FEI Tecnai, Hillsboro, OR, USA). TEM images were captured at an acceleration voltage of 200 kV. The high-resolution TEM images allowed for the detailed observation of cellular and tissue structures at the nanoscale, including cell membranes and mitochondria.

### Detection of mitochondrial membrane potential (ΔΨm) using JC-1 assay

Mitochondrial membrane potential (ΔΨm) was assessed using the JC-1 fluorescence probe to evaluate the impact of SARS-CoV-2 S protein expression on lung epithelial cell mitochondrial function. Briefly, cells were seeded in a 96-well plate and transfected once 40 % confluence was reached. After treatment, cells were washed three times with PBS and processed according to the manufacturer's protocol (Beyotime, Shanghai, China). Fluorescence signals were measured using a multifunctional microplate reader (CLARIOstar), with excitation/emission wavelengths set at 490 nm/530 nm for JC-1 monomers and 525 nm/590 nm for JC-1 aggregates.

### Detection of mitochondrial reactive oxygen species (ROS) by flow cytometry (FCM)

MitoSOX Red dye (Thermo Fisher, Waltham, MA, USA) was used to detect mitochondrial ROS levels to assess the impact of SARS-CoV-2 S protein expression on mitochondrial oxidative stress. Cells were seeded in a 6-well plate and transfected when they reached 40 % confluence. After treatment, cells were washed three times with PBS, digested with trypsin, and collected by centrifugation at 600 × g for 10 min, discarding the supernatant. The cell pellet was resuspended in serum-free medium, and MitoSOX dye (prepared according to the kit instructions) was added. Cells were incubated at 37 °C in the dark for 30 min. Following staining, cells were washed three times, filtered, and analyzed using a Beckman Coulter flow cytometer (Beckman Coulter, Brea, CA, USA).

### Evaluation of glycolysis metabolism reprogramming induced by SARS-CoV-2 S protein

The impact of SARS-CoV-2 S protein expression on glucose uptake in lung epithelial cells was evaluated using the 2-NBDG fluorescent glucose probe (MedChemExpress, Monmouth Junction, NJ, USA). Cells were seeded in a 6-well plate and transfected at 40 % confluence. After treatment, cells were washed three times with PBS and incubated with the 50 μmol/L 2-NBDG working solution at 37 °C in the dark for 1 h. Following incubation, cells were digested with trypsin, collected by centrifugation at 600 × g for 10 min, and the supernatant was discarded. The cell pellet was resuspended in PBS, filtered, and analyzed for fluorescence using a Beckman Coulter flow cytometer.

After the indicated treatment, cells were washed three times with PBS, scraped, and homogenized for 5 min on ice. The activities of hexokinase (HK) and pyruvate kinase (PK) were measured according to the manufacturer's protocol (Boxbio, Beijing, China). The OD value was measured at 340 nm using a CLARIOstar multifunctional microplate reader. Lactic acid production in cells was measured according to the manufacturer's protocol (Beyotime, Shanghai, China), and the OD value was measured at 530 nm using a CLARIOstar multifunctional microplate reader. ATP content was measured according to the manufacturer's protocol (Beyotime, Shanghai, China). Luminescence signals were measured using a CLARIOstar multifunctional microplate reader.

### Statistical analysis

Statistical analysis was carried out using the Statistical Package for Social Sciences (SPSS), version 20.0 (IBM Corp, Armonk, NY, USA). Data are presented as mean ± standard deviation (SD), derived from a minimum of three independent experiments. Two-tailed unpaired Student's *t*-test was employed for comparisons between two groups. When comparing multiple groups, one-way analysis of variance (ANOVA) followed by Dunnett's *t*-test was used. Significance levels were denoted as * for *P* < 0.05.

## Results

### SARS-CoV-2 S protein-induced pyroptosis and inflammatory responses in lung epithelial cells *in vitro*

**Fig. 1:**
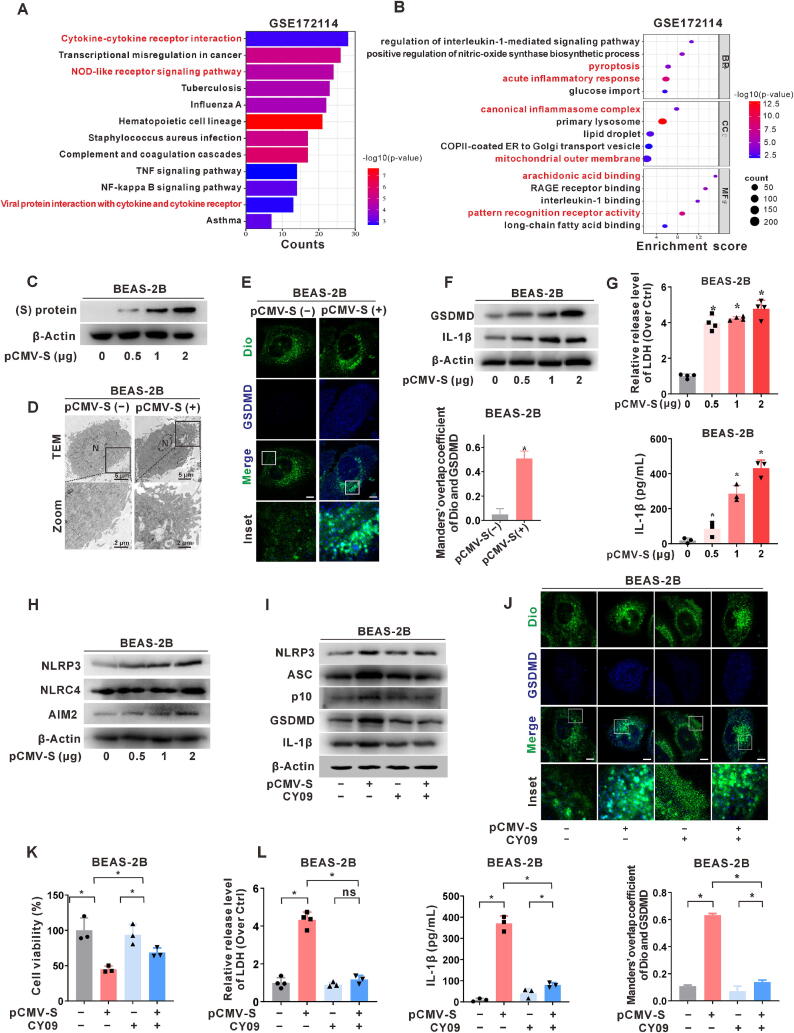
**SARS-CoV-2 S protein-induced pyroptosis and inflammatory responses in lung epithelial cells. (A-B)** Data retrieved from the GEO database (GSE172114) based on whole blood samples from patients with COVID-19 (n = 53) were screened for analysis. (**A)** KEGG pathway annotation was performed to assess NLRs-related inflammatory pathways associated with differentially expressed genes (DEGs). (**B)** Gene ontology (GO) enrichment analysis of DEGs was conducted to elucidate the functional implications of the observed gene expression changes. (**C**–**H)** BEAS-2B cells were transfected with varying doses (0.5–2 μg) of the pCMV3-SARS-CoV-2 S (pCMV-S) plasmid for 24 h. (**C)** Western blotting (WB) was employed to confirm the expression of the SARS-CoV-2 S protein in cells. (**D)** Transmission electron microscopy (TEM) revealed membrane rupture in the cells, indicating cellular morphological changes. N, nucleus. Scale bar, 5 μm for the gobal view and 2 μm for the zoom view. (**E)** An immunofluorescence (IF) assay showed the co-localization of Dio (green) and GSDMD (blue) using confocal microscopy (left), illustrating fluorescence intensity alterations. Scale bar, 10 μm. Manders' overlap coefficient quantification for Dio and GSDMD co-localization of cells is presented in a bar graph (right). (**F)** WB analysis of GSDMD and IL-1β protein expression. (**G)** LDH release assay (upper) and ELISA (lower) were used to assess plasma membrane rupture and IL-1β release levels in the supernatant, respectively. (**H)** WB analysis evaluated the expression of NLRP3, NLRC4, and AIM2 proteins, indicating inflammasome levels. (**I-L)** CY09 was treated in S-expressing cells. BEAS-2B cells were pre-treated with CY09 (20 μM) for 3 h, followed by transfection with pCMV-S (1 μg) for 24 h or not. (**I)** WB was used to examine the expression of proteins related to NLRP3 inflammasome activation (NLRP3, ASC, and p10) and pyroptosis (GSDMD and IL-1β). (**J)** IF assay of Dio (green) and GSDMD (blue) was used to assess cellular membrane changes (upper). Scale bar, 10 μm. Manders' overlap coefficient quantification for Dio and GSDMD co-localization in cells is presented in a bar graph (lower). (**K)** Cell viability was measured using the MTT assay. (**L)** LDH release assay (left) and ELISA (right) were used to measure plasma membrane rupture and IL-1β release levels in the supernatant, respectively. *, *P* < 0.05, compare to the control group or corresponding group. (For interpretation of the references to colour in this figure legend, the reader is referred to the web version of this article.)

To investigate the alterations in inflammation-related genes throughout the progression of COVID-19, a comprehensive analysis was performed using the GSE172114 database. Enrichment analysis revealed that the key enriched pathways included the NOD-like receptor (NLR) pathway, the tumor necrosis factor α (TNF-α) pathway, and the nuclear factor-kappa B signaling pathway (Fig. 1A). These findings highlight the robust immune and inflammatory responses induced by SARS-CoV-2 infection. Moreover, GO analysis indicated that genes differentially expressed in patients with severe COVID-19 were enriched in pathways such as IL-1 signaling, pyroptosis, classical inflammasome activity, and glucose uptake compared to non-critical patients (Fig. 1B). These findings highlight the potent pyroptosis and inflammasome-activated reactions induced by SARS-CoV-2 infection, which may be involved in COVID-19-related lung inflammatory damage.

Previous studies have demonstrated that SARS-CoV-2 infection can induce a hyperinflammatory syndrome, characterized by the overexpression of pro-inflammatory factors. Notably, in patients with COVID-19, pro-inflammatory cytokines, including IL-1β and IL-6, demonstrated a strong correlation with disease severity. In the current study, we focused on assessing the impact of SARS-CoV-2 protein expression on lung epithelial cells, with particular emphasis on the S protein. We used recombinant plasmids, pCMV3-SARS-CoV-2 S (pCMV-S), pTT5-SARS-CoV-2(M), and pTT5-SARS-CoV-2(N), generated via human codon optimization. These plasmids enabled the transient transfection of human lung epithelial BEAS-2B cells, thereby establishing *in vitro* models for the expression of the S, M, and N proteins. The mRNA levels of inflammatory factors and NLR family members, as well as secretion of IL-1β, were significantly higher in BEAS-2B cells transfected with the SARS-CoV-2 S protein-expressing plasmid compared to cells transfected with other structural protein-coding plasmids (Fig. S1A-C). To investigate the mechanism underlying the inflammatory response induced by S protein expression in lung epithelial cells, we conducted a series of targeted interventions. The viability of BEAS-2B cells expressing the S protein decreased significantly in a dose-dependent manner compared to that of the control group (Fig. S1D). In particular, BEAS-2B cells transfected with pCMV-S were treated with Fer-1 (a ferroptosis inhibitor), VX765 (a pyroptosis inhibitor), and Nec-1 (a necroptosis inhibitor). Treatment with VX765 resulted in a significant reduction in IL-1β release and rescued cell viability compared to the non-intervened S protein expression group (Fig. S1E-F). These results indicated that S protein expression not only diminished BEAS-2B cell viability but also promoted IL-1β secretion, implicating the involvement of pyroptosis in this process within lung epithelial cells. Therefore, the cellular model of S protein expression (S-expressing group) was investigated in detail (Fig. 1C and Fig. S1G). Distinct cytoplasmic membrane disruption and release of cytoplasmic contents were observed in the S-expressing lung epithelial cells using TEM, distinguishing them from the control cells (Fig. 1D and Fig. S1H). Immunofluorescence (IF) analysis revealed that compared to control cells, the S-expressing group displayed higher gasdermin D (GSDMD) fluorescence signals and enhanced GSDMD co-localization with the Dio-labeled cell membrane (Fig. 1E and Fig. S1I).

Subsequent analysis showed that BEAS-2B and A549 cells expressing the S protein exhibited higher levels of pyroptosis-related proteins, including GSDMD and IL-1β (Fig. 1F and Fig. S1J). Notably, we observed a dose-dependent increase in lactate dehydrogenase (LDH) release and IL-1β secretion in the S-expressing group compared to the control group (Fig. 1G and Fig. S1K). These results indicated that the S protein expression induces pyroptosis in lung epithelial cells. This process leads to cell membrane perforation and the subsequent release of inflammatory mediators. To investigate the regulatory role of inflammasomes in S protein-induced pyroptosis in lung epithelial cells, we assessed the expression of several key inflammasomes (Fig. S1B). BEAS-2B and A549 cells expressing the S protein showed a significant increase in NLRP3 protein expression (Fig. 1H and Fig. S1L).

Additionally, we established an inhibition model of the NLRP3 inflammasome in lung epithelial cells using CY09, a small-molecule inhibitor known for its ability to limit NLRP3 activity [17,32]. Compared to the S protein-expressing cells, cells treated with CY09 in conjunction with S-expression exhibited a noticeable reduction in the activation of NLRP3 inflammasome- and pyroptosis-related proteins (Fig. 1I and Fig. S1M). Additionally, the results of IF indicated that the CY09-intervened S-expressing group displayed a weakened GSDMD fluorescence signal and reduced co-localization with Dio-labeled cell membranes in BEAS-2B cells, compared to the S protein-expressing group (Fig. 1J). Moreover, the viability of lung epithelial cells was partially rescued in the CY09-intervened S-expressing group (Fig. 1K and Fig. S1N), while the levels of LDH release and IL-1β secretion in the cell supernatant decreased notably (Fig. 1L and Fig. S1O). These results suggested that targeting the inhibition of NLRP3 inflammasome activation may significantly reduce pyroptosis and control inflammatory responses in lung epithelial cells expressing the SARS-CoV-2 S protein.

### Regulation of mitochondrial quality control and metabolic reprogramming by SARS-CoV-2 S protein expression in lung epithelial cells

**Fig. 2:**
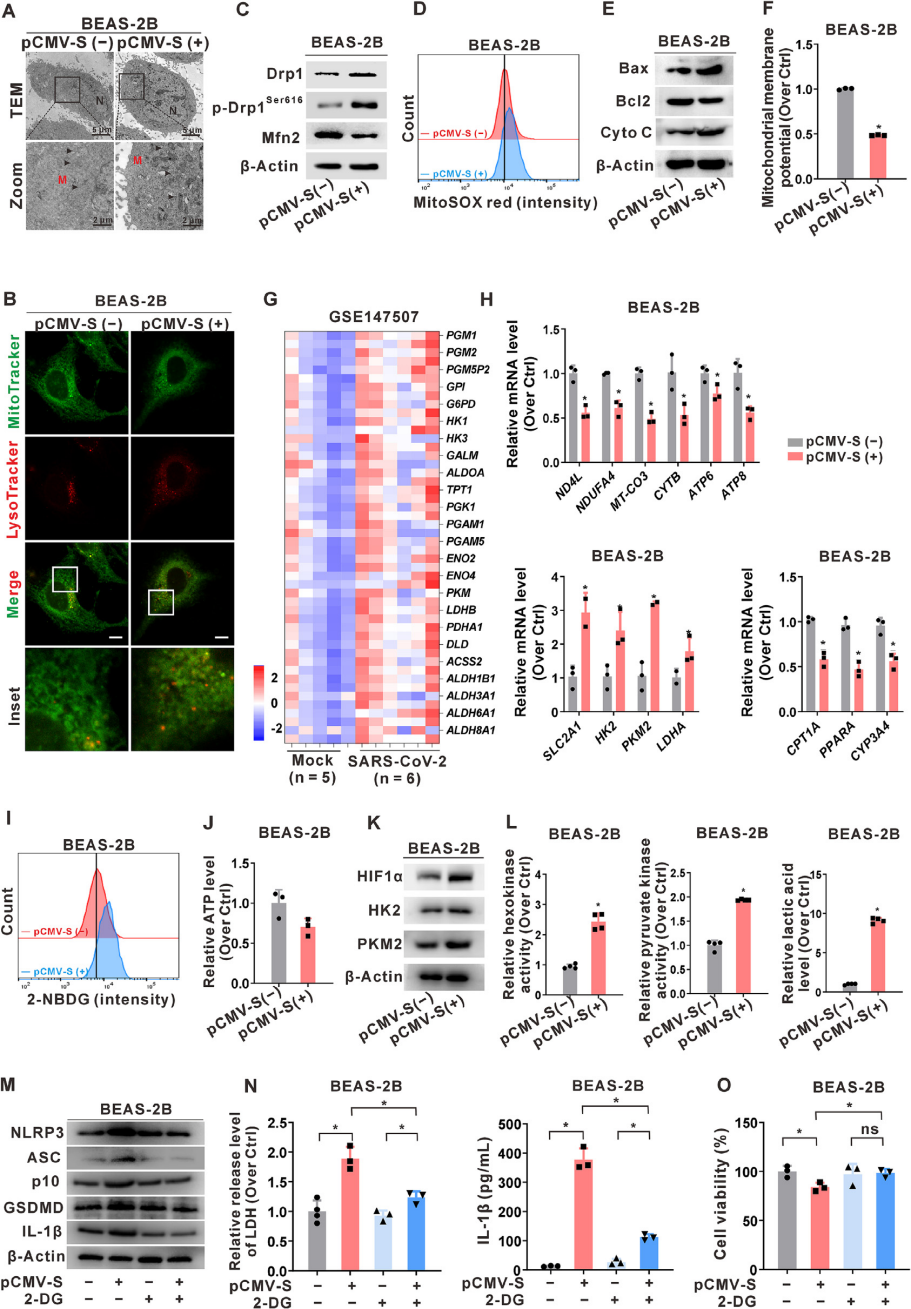
**Regulation of SARS-CoV-2 S protein expression on mitochondrial quality control and metabolic reprogramming in lung epithelial cells. (A-F, H-L)** BEAS-2B cells were transfected with pCMV3-SARS-CoV-2 S (pCMV-S, 1 μg for 24 h) to induce the S protein expression. (**A)** TEM observation of mitochondrial morphology in cells. N, nucleus. M, mitochondria. Scale bar, 5 μm for the gobal view and 2 μm for the zoom view. (**B)** IF assay of MitoTracker (green) and LysoTracker (red) was used to assess the intensity and co-localization in cells. Scale bar, 10 μm. (**C)** WB analysis of mitochondrial dynamics-associated proteins (Drp1, p-Drp1^Ser616^, and Mfn2). (**D)** Flow cytometry (FCM) assay of mitochondrial ROS levels using MitoSOX (red) in cells. (**E)** WB analysis of mitochondrial damage-related proteins (Bax, Bcl2, and Cyto C). (**F)** JC-1 assay of mitochondrial membrane potential. (**G)** The GEO dataset (GSE147507) from SARS-CoV-2-infected A549 cell samples (n = 6) was screened, while mock samples (n = 5) used as controls. The heatmap depicts the DEGs of glycolysis-related genes. (**H)** Relative mRNA levels of mitochondrial energy metabolism-related genes in cells. (**I)** FCM assay of glucose flux using fluorescent glucose analog 2-NBDG in cells. (**J)** Detection of ATP content in cells. (**K)** WB analysis of glycolysis-related proteins (HIF1α, HK2, and PKM2). (**L)** Levels of glycolysis rate-limiting enzymes (hexokinase and pyruvate kinase) activities and glycolysis product lactic acid. **(M−O)** BEAS-2B cells were pre-treated with the hexokinase inhibitor 2-deoxy-D-glucose (2-DG, 20 μM for 3 h), followed by transfection with pCMV-S (1 μg for 24 h) or mock transfection. **(M)** WB analysis of NLRP3 inflammasome activation (NLRP3, ASC, and p10) and pyroptosis-related proteins (GSDMD and IL-1β). (**N)** LDH release assay and ELISA detection of IL-1β release levels in the supernatant. (**O)** Cell viability was measured using the MTT assay. *, *P* < 0.05, compare to the control group or corresponding group. (For interpretation of the references to colour in this figure legend, the reader is referred to the web version of this article.)

Given that NLRP3 inflammasome activation is typically associated with mitochondrial damage, we investigated the impact of S protein expression on the regulation of mitochondrial function in lung epithelial cells [[33], [34], [35]]. TEM revealed notable abnormalities in mitochondrial morphology in lung epithelial cells of the S protein-expressing group compared to the control group, including widened gaps within the mitochondrial cristae and increased density, resulting in a distinctive zebra-like (striped) appearance (Fig. 2A and Fig. S2A). IF analysis showed increased intensities of MitoTracker, denoting fragmentation of mitochondria, and LysoTracker, marking lysosomes and their co-localization with MitoTracker. These findings were indicative of mitophagy in lung epithelial cells expressing the S protein, unlike in the control group (Fig. 2B and Fig. S2B). Furthermore, compared to the control group, the S-expressing group exhibited increased levels of Drp1 and p-Drp1^Ser616^ but decreased levels of Mfn2 (Fig. 2C and Fig. S2C). FCM analysis of MitoSOX staining demonstrated increased levels of mitochondrial ROS in S-expressing cells, as indicated by enhanced fluorescence intensity (Fig. 2D and Fig. S2D).

Additionally, increased levels of Bax and release of cytochrome *C* were observed in S-expressing cells compared to those in the control group (Fig. 2E and Fig. S2E). Similarly, loss of mitochondrial membrane potential was detected in the S-expressing cells compared to the control cells (Fig. 2F and Fig. S2F). Collectively, these results suggest that S protein expression disrupts mitochondrial quality control in lung epithelial cells. Given the central role of mitochondria in energy metabolism, the effect of S protein expression on mitochondrial metabolism was also examined. Analysis of the GEO database from SARS-CoV-2-infected A549 cell samples (GSE147507) revealed upregulation of glycolysis-related genes compared to mock samples (Fig. 2G).

Similarly, in S protein-expressing BEAS-2B cells, the mRNA levels of genes related to mitochondrial oxidative phosphorylation and the electron transport chain were significantly reduced, whereas the mRNA levels of glycolysis-related genes were significantly elevated (Fig. 2H). Cellular glucose flux was evaluated using the fluorescent glucose analog 2-NBDG. FCM analysis revealed a significant increase in glucose uptake capacity in lung epithelial cells expressing the S protein compared to the control cells, indicating a shift towards alternative glucose metabolism pathways other than mitochondrial oxidative phosphorylation (Fig. 2I and Fig. S2G). Furthermore, the ATP content in the S-expressing cells was lower than that in control cells (Fig. 2J and Fig. S2H). Subsequent investigations demonstrated that the S protein-expressing cells exhibited significantly increased protein and activity levels of the glycolysis rate-limiting enzymes, hexokinase and the pyruvate kinase, along with elevated levels of the glycolysis metabolite lactic acid (Fig. 2K-L and Fig. S2I-J). These findings suggest that the mitochondrial respiratory chain function is inhibited in S protein-expressing lung epithelial cells, prompting a metabolic shift towards glycolysis.

Recent studies have shown that SARS-CoV-2 infection contributes to inflammatory injury by inducing a glycolytic shift in infected cells [20]. Therefore, we investigated the role of glycolytic metabolic reprogramming in S protein expression-induced NLRP3 inflammasome activation and subsequent pyroptosis in lung epithelial cells. We established a glycolysis inhibition model in lung epithelial cells using the hexokinase inhibitor 2-deoxy-D-glucose (2-DG, 20 μM). Compared to control cells, the expression levels of proteins related to NLRP3 inflammasome activation and pyroptosis were notably reduced in 2-DG-treated S-expressing cells (Fig. 2M and Fig. S2K). This reduction was accompanied by a significant decrease in LDH release and IL-1β secretion in the supernatant (Fig. 2N and Fig. S2L), indicating the rescue of cell damage while cell viability increased in the 2-DG-treated group (Fig. 2O and Fig. S2M). These results suggest that S protein expression modulates NLRP3 inflammasome activation and pyroptosis by reprogramming glycolysis in lung epithelial cells.

### SARS-CoV-2 S protein-induced COX-2 regulates mitochondrial quality control and metabolic reprogramming, inhibiting pyroptosis

**Fig. 3:**
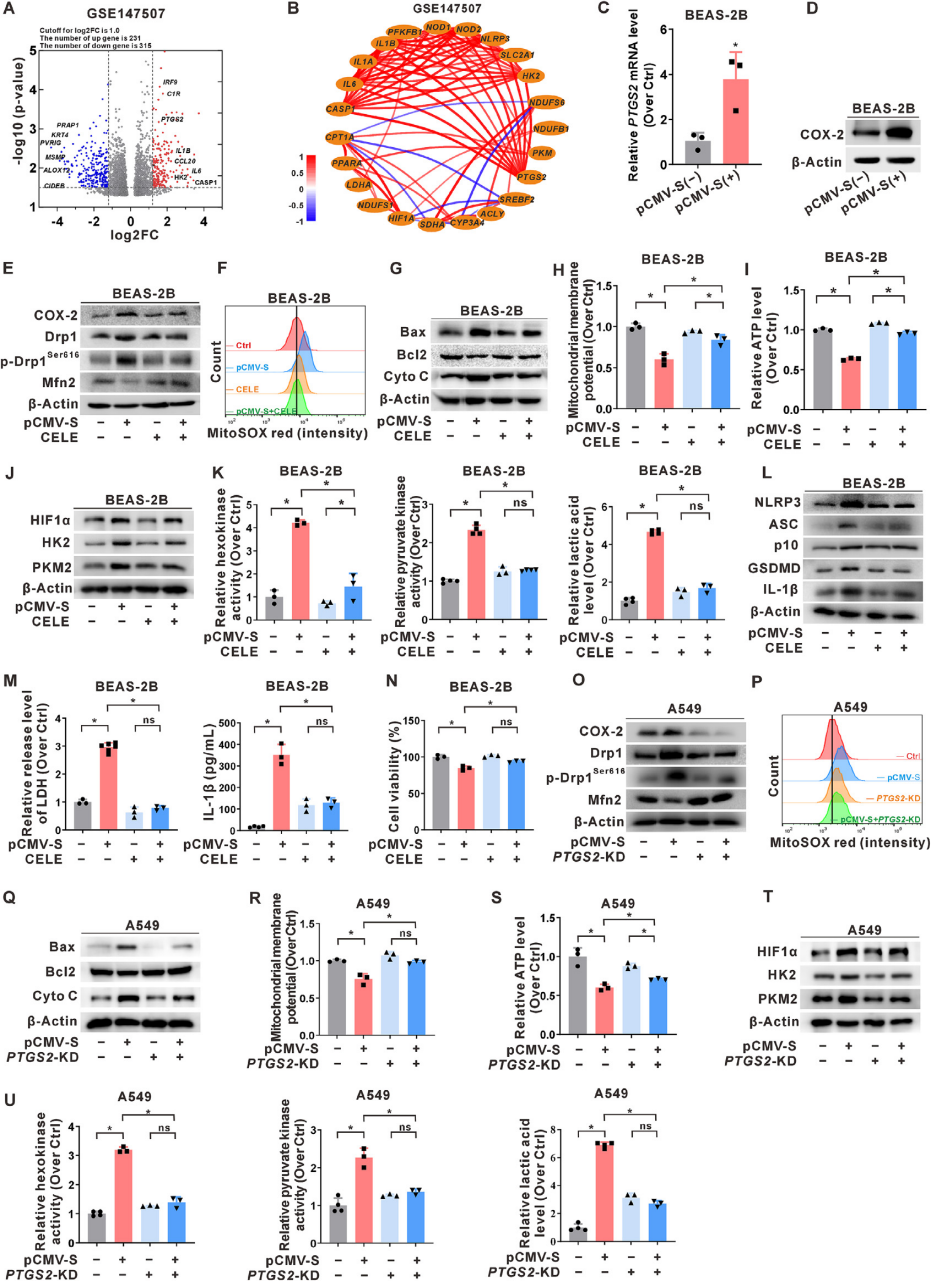
**SARS-CoV-2 S protein-induced COX-2 regulates mitochondrial quality control and metabolic reprogramming, inhibiting pyroptosis. (A-B)** The GEO database (GSE147507) was screened and analyzed from SARS-CoV-2-infected A549 cells. (**A)** Volcano plot displaying DEGs. (**B)** Correlation coefficient network diagram of DEGs depicting the relationship between *PTGS2* and inflammation- or metabolism-related genes. (**C-N)** BEAS-2B cells were transfected with pCMV3-SARS-CoV-2 S (pCMV-S, 1 μg for 24 h) and pre-treated with celecoxib (CELE, 40 μM for 3 h) or not. Levels of *PTGS2* mRNA **(C)** and COX-2 protein **(D)** were shown. (**E)** WB analysis of COX-2 and mitochondrial dynamics proteins (Drp1, p-Drp1^Ser616^, and Mfn2). (**F)** FCM assay of mitochondrial ROS levels using MitoSOX (red) in cells. (**G)** WB analysis of mitochondrial damage-related proteins (Bax, Bcl2, and Cyto C). (**H)** JC-1 assay of mitochondrial membrane potential. (**I)** Detection of ATP content in cells. (**J)** WB analysis of glycolysis-related proteins (HIF1α, HK2, and PKM2). (**K)** Levels of glycolysis rate-limiting enzymes (hexokinase and pyruvate kinase) activities and glycolysis product lactic acid. (**L)** WB analysis of NLRP3 inflammasome activation (NLRP3, ASC, and p10) and pyroptosis-related proteins (GSDMD and IL-1β). (**M)** LDH release assay and ELISA detection of IL-1β release levels in the supernatant. (**N)** Cell viability was measured using the MTT assay. (**O-U)** A stable COX-2 knockdown A549-*PTGS2*-KD cell was constructed using a Cas9-*PTGS2* recombinant plasmid. (**O)** WB analysis of COX-2 and mitochondrial dynamics proteins (Drp1, p-Drp1^Ser616^, and Mfn2). (**P)** FCM assay of mitochondrial ROS levels using MitoSOX (red) in cells. (**Q)** WB analysis of mitochondrial damage-related proteins (Bax, Bcl2, and Cyto C). (**R)** JC-1 assay of mitochondrial membrane potential. (**S)** Detection of ATP content in cells. (**T)** WB analysis of glycolysis-related proteins (HIF1α, HK2, and PKM2). (**U)** Levels of glycolysis rate-limiting enzymes (hexokinase and pyruvate kinase, left and middle) activities and glycolysis product lactic acid (right). *, *P* < 0.05, compare to the control group or corresponding group. (For interpretation of the references to colour in this figure legend, the reader is referred to the web version of this article.)

Among the COX enzymes, COX-1 is constitutively expressed in most tissues, whereas COX-2 (encoded by the *PTGS2* gene) is typically induced in larger quantities during pathological states, contributing to regulation of inflammation [17]. Analysis of the GSE147507 database from SARS-CoV-2-infected A549 cells revealed upregulation of *PTGS2* and inflammation-related genes in virus-infected lung epithelial cells (Fig. 3A). Furthermore, correlation coefficient network analysis demonstrated a significant positive correlation between *PTGS2* and genes related to inflammation and glycolysis (Fig. 3B). Consequently, both COX-2 gene and protein expression were increased in lung epithelial cells expressing the S protein (Fig. 3C-D, and Fig. S3A). SARS-CoV-2 infection hijacks host cell metabolism to meet its replication demands, prompting a metabolic reprogramming from the tricarboxylic acid cycle and oxidative phosphorylation to glycolysis. This shift provides the energy necessary for viral replication, immune evasion, and cellular damage [[36], [37], [38]]. To further investigate the role of COX-2 in mitochondrial damage and metabolic reprogramming in S protein-expressing lung epithelial cells, we established an intervention model using the COX-2 selective inhibitor, celecoxib (CELE). The results indicated that the levels of mitochondrial fission-related proteins Drp1 and p-Drp1^Ser616^ decreased, while the level of Mfn2, which is related to mitochondrial fusion, increased in CELE-treated S-expressing cells (Fig. 3E and Fig. S3B). FCM analysis revealed a reduction in the fluorescence intensity of MitoSOX (Fig. 3F and Fig. S3C), decreased level of Bax and release of cytochrome *C* (Fig. 3G and Fig. S3D), repolarized mitochondrial membrane potential, and increased ATP content in CELE-treated S-expressing cells (Fig. 3H-I, and Fig. S3E). These results indicate that COX-2 is involved in the regulation of S protein-induced mitochondrial dysfunction and that targeting COX-2 could rescue mitochondrial quality control in lung epithelial cells.

Moreover, the protein expression and activity of the rate-limiting enzymes of glycolysis, hexokinase and pyruvate kinase, were decreased in S-expressing BEAS-2B and A549 cells treated with CELE, whereas lactic acid content was also reduced in these cells (Fig. 3J-K, and Fig. S3F-G). These findings suggest that targeting COX-2 could rescue the heightened glycolytic activity in lung epithelial cells expressing the S protein. We further investigated the role of COX-2-related mitochondrial metabolic reprogramming in inflammation induced by S protein expression. Downregulation of NLRP3 and inflammasome activation, as well as pyroptosis-related proteins, was observed in the CELE intervention group (Fig. 3L and Fig. S3H), while LDH release and IL-1β secretion in the supernatant were significantly reduced, along with recovery of cell viability (Fig. 3M-N, and Fig. S3I-J). These results suggest that COX-2 intervention could effectively inhibit NLRP3 inflammasome activation and pyroptosis induced by S protein expression in lung epithelial cells.

To investigate the role of COX-2 in promoting mitochondrial glycolytic metabolic reprogramming induced by S protein expression, we created stable Cas9-based COX-2 knockdown A549-*PTGS2*-KD cells (Fig. S3K). Compared to control cells in the S protein-expressing group, A549-*PTGS2*-KD cells expressing the S protein exhibited decreased expression of Drp1 and p-Drp1^Ser616^, increased expression of Mfn2, and reduced expression of COX-2 (Fig. 3O). FCM analysis revealed that mitochondrial ROS levels (Fig. 3P), Bax levels, and release of cytochrome *C* were reduced (Fig. 3Q), mitochondrial membrane potential was repolarized (Fig. 3R), and ATP content was upregulated in S-expressing COX-2 knockdown cells (Fig. 3S). These findings suggest that downregulation of COX-2 can mitigate mitochondrial quality control disruption in S-expressing lung epithelial cells. Further investigation demonstrated that COX-2 knockdown decreased the protein levels and activities of glycolytic rate-limiting enzymes and reduced lactic acid content (Fig. 3T-U). These results indicate that SARS-CoV-2 S protein-induced COX-2 upregulation is involved in NLRP3 inflammasome activation and pyroptosis by promoting glycolytic metabolic reprogramming. Targeting COX-2 could potentially remodulate mitochondrial metabolism in lung epithelial cells.

### Modulation of mitochondrial COX-2 localization and interaction with HK2 by SARS-CoV-2 S protein in lung epithelial cells

**Fig. 4:**
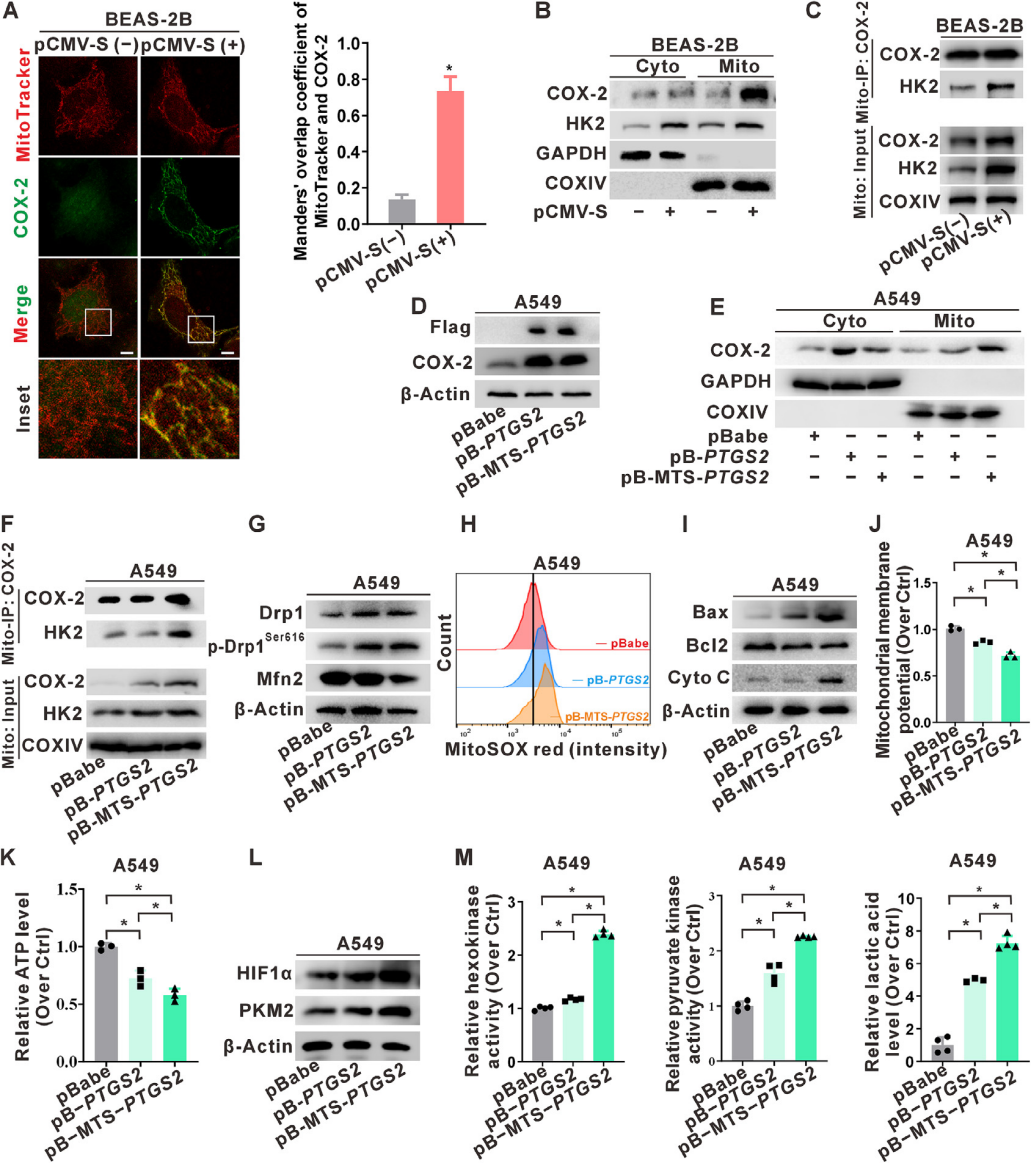
**Modulation of mitochondrial COX-2 localization and interaction with HK2 by expressing SARS-CoV-2 S protein in lung epithelial cells*.* (A-C)** BEAS-2B cells transfected with pCMV-S (1 μg) for 24 h or mock transfection. (**A)** IF assay observation of MitoTraker (red) and COX-2 (green) by confocal microscopy (left). Manders' overlap co-efficient quantification for MitoTracker and COX-2 co-localization in cells is presented in a bar graph (right). Scale bar, 10 μm. (**B)** WB analysis of COX-2 expression in cytoplasmic (Cyto) and mitochondrial (Mito) fractions. (**C)** Mitochondrial fractions were immunoprecipitated with an anti-COX-2 antibody using Mito-IP assay, and the expression of HK2 was detected by WB. (**D-L)** Plasmids pB-*PTGS2* and pB-MTS-*PTGS2* were constructed and used to establish stable cells with COX-2 overexpression, while the pBabe vector was used as a control. (**D)** WB detection of COX-2 and Flag protein levels. (**E)** Protein levels of COX-2 expression in cytoplasmic (Cyto) and mitochondrial (Mito) fractions were detected by WB. (**F)** Mito-IP with an anti-COX-2 antibody, and the expression of HK2 was detected by WB. (**G)** WB analysis of mitochondrial dynamics proteins (Drp1, p-Drp1^Ser616^, and Mfn2). (**H)** FCM assay of mitochondrial ROS levels using MitoSOX (red) in cells. (**I)** WB analysis of mitochondrial damage-related proteins (Bax, Bcl2, and Cyto C). (**J)** JC-1 assay of mitochondrial membrane potential. (**K)** Detection of ATP content in cells. (**L)** WB analysis of glycolysis-related proteins (HIF1α and PKM2). (**M)** Levels of glycolysis rate-limiting enzymes (hexokinase and pyruvate kinase) activities and glycolysis product lactic acid. *, *P* < 0.05, compare to the control group or corresponding group. (For interpretation of the references to colour in this figure legend, the reader is referred to the web version of this article.)

To investigate the correlation between *PTGS2* and the genes encoding the rate-limiting enzymes of glycolysis (*HK1*, *HK2*, and *HK3*), we analyzed the GEO databases. The analysis revealed a significant positive correlation between the relative expression levels of *PTGS2* and *HK2* in patients with COVID-19 (GSE172114: *r* = 0.4659, *P* < 0.05) and in SARS-CoV-2 infected A549 cells (GSE147507: *r* = 0.8878, *P* < 0.05) (Fig. S4A-B). IF demonstrated that, compared to control cells, COX-2 expression and distribution were intensified in S-expressing cells, with increased co-localization of COX-2 and MitoTracker-labeled mitochondria (Fig. 4A). Moreover, the protein levels of COX-2 and HK2 in the mitochondrial fraction were higher in S-expressing BEAS-2B cells than in control cells (Fig. 4B). Furthermore, Mito-IP analysis showed elevated interaction between COX-2 and HK2 in S-expressing cells (Fig. 4C). These findings suggest that S protein expression enhances the interaction between COX-2 and HK2 in the mitochondria.

To validate the functionality of mitochondrial COX-2 and its regulation, we used pB-*PTGS2* and pB-MTS-*PTGS2* plasmids to establish stable COX-2 overexpression and enhanced mitochondrial localization in cell models. Western blotting indicated a substantial increase in COX-2 expression, especially in the mitochondrial fraction of A549-MTS-*PTGS2* cells (Fig. 4D-E). HK2 can be induced by various environmental factors and recruited to the outer mitochondrial membrane to participate in glycolysis and regulation of mitochondrial function [39]. The mitochondrial fraction was extracted for Mito-IP analysis, as shown in Fig. 4F. The interaction between COX-2 and HK2 was increased in the mitochondrial fractions of COX-2-overexpressing cells, especially in A549-MTS-*PTGS2* cells. Furthermore, in A549-MTS-*PTGS2* cells, the levels of Drp1 and p-Drp1^Ser616^ increased, whereas levels of Mfn2 decreased (Fig. 4G). Compared to A549-pBabe and A549-*PTGS2* cells, the fluorescence intensity of MitoSOX (Fig. 4H), the level of Bax, and the release of cytochrome *C* increased (Fig. 4I); mitochondrial membrane potential was depolarized, and ATP content decreased (Fig. 4J-K). Further investigations revealed increased protein levels and activities of hexokinase and pyruvate kinase (Fig. 4L), along with an increase in lactic acid content (Fig. 4M) in the A549-MTS-*PTGS2* cells. These results imply that SARS-CoV-2 S protein expression induces mitochondrial COX-2 translocation and its interaction with HK2. Moreover, the mitochondrial targeting of COX-2 promotes reprogramming of mitochondrial quality control-mediated glycolytic metabolism.

### Regulation of mitochondrial COX-2^Cys555^ palmitoylation by SARS-CoV-2 S protein-induced DHHC5 and its subsequent interaction with HK2

**Fig. 5:**
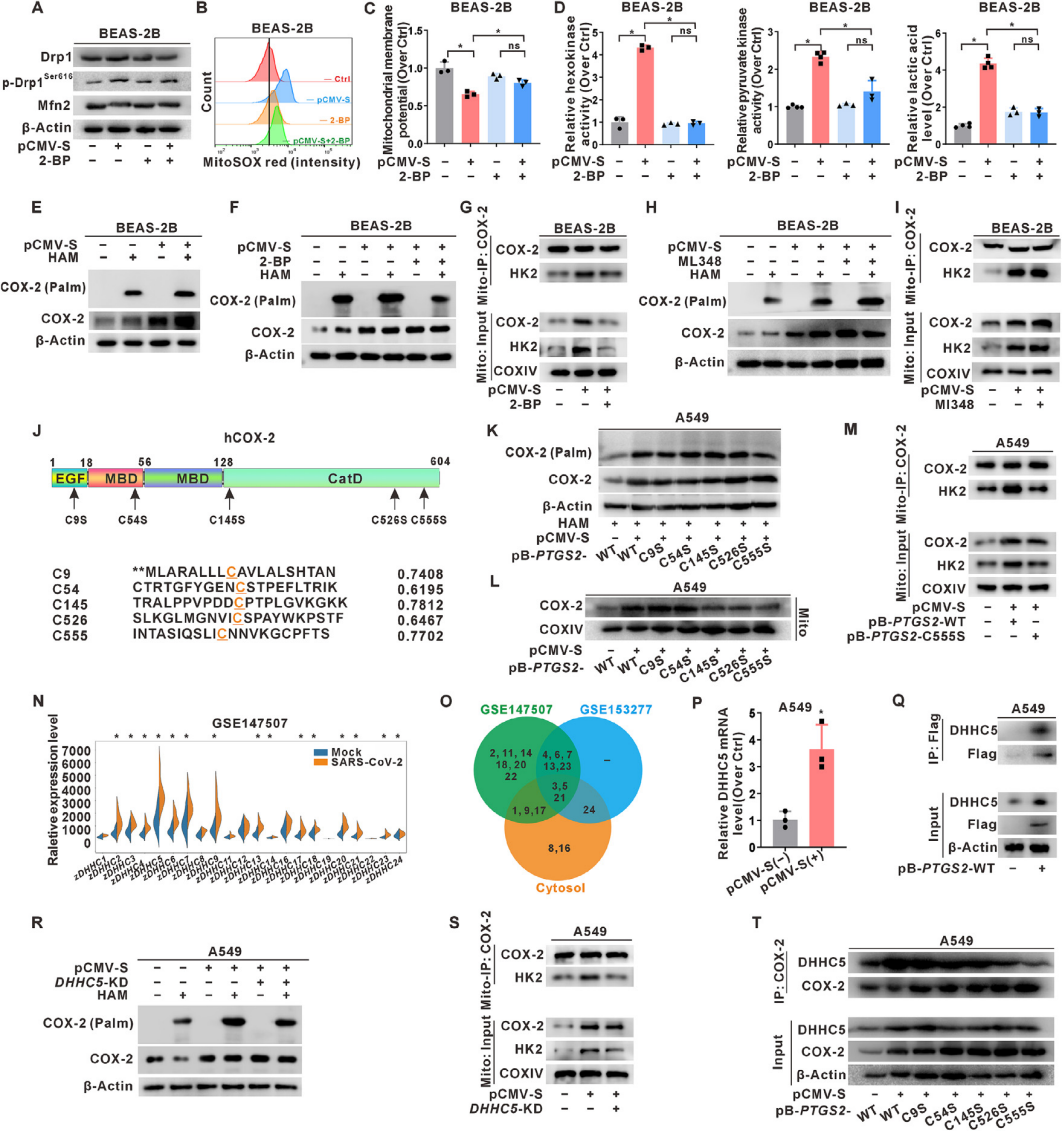
**Regulation of mitochondrial COX-2^Cys555^ palmitoylation by SARS-CoV-2 S protein-induced DHHC5 and its subsequent interaction with HK2. (A-D, F)** BEAS-2B cells were pre-treated with a non-specific palmitoylation inhibitor, 2-BP (40 μM, for 3 h), followed by transfection with pCMV-S (1 μg for 24 h) or mock transfection. (**A)** WB analysis of mitochondrial dynamics proteins (Drp1, p-Drp1^Ser616^, and Mfn2). (**B)** FCM assay of mitochondrial ROS levels using MitoSOX (red) in cells. (**C)** JC-1 assay of mitochondrial membrane potential. (**D)** Levels of glycolysis rate-limiting enzymes (hexokinase and pyruvate kinase) activities and glycolysis product lactic acid. (**E-F)** The acyl-resin-assisted capture (Acyl-RAC) assay was performed to detect COX-2 palmitoylation and to evaluate the effect of 2-BP on this modification. Hydroxylamine (HAM) was used for the selective cleavage of thioester bonds to capture palmitoylated proteins in the Acyl-RAC assay, with Tris serving as a negative control. (**G)** Mito-IP was performed using an anti-COX-2 antibody, and the expression of HK2 was detected by WB. **(H-I)** BEAS-2B cells were treated with the depalmitoylation inhibitor ML348 (10 μM, for 24 h). (**H)** The effects of ML348 on COX-2 palmitoylation were detected by Acyl-RAC assay. (**I)** Mito-IP was performed using an anti-COX-2 antibody, and the expression of HK2 was detected by WB. (**J)** A schematic view of COX-2 (full-length) and the sites of *S*-palmitoylation-deficient COX-2 variants. The palmitoylation sites were predicted using the CSS-palm 2.0 predictor (http://csspalm.biocuckoo.org/). The scores represented the probability for palmitoylation for different COX-2 variants. A potential site is considered when the score is > 0.6. (**K)** WB detection of palmitoylated COX-2 in A549 cells expressing wild-type (WT) or *S*-palmitoylation-deficient COX-2 variants (*PTGS2*-C5S, −C54S, −C145S, −C526S, and −C555S) using the Acyl-RAC assay with HAM treatment. (**L)** WB detection of the COX-2 levels in mitochondrial fractions. (**M)** Mito-IP with an anti-COX-2 antibody, and the expression of HK2 was detected by WB. (**N)** The GEO database (GSE147507) of SARS-CoV-2-infected A549 cell samples was screened to analyze the expression of *DHHC* family genes. (**O)** Differential expression and cytoplasmic distribution of *DHHC* family genes in GEO databases (GSE147507 and GSE153277) were presented. (**P)** mRNA levels of *DHHC5* in cells. (**Q)** Whole cell lysates were immunoprecipitated with an anti-Flag antibody, and the expression of DHHC5 was detected by WB. (**R**-**S)** A stable DHHC5 knockdown A549-*DHHC5*-KD cell line was constructed using a Cas9-*DHHC5* recombinant plasmid. **(R)** Acyl-RAC assay of palmitoylated COX-2. (**S)** Mito-IP with an anti-COX-2 antibody, and the expression of HK2 was detected by WB. (**T)** Whole cell lysates were immunoprecipitated with an anti-COX-2 antibody, and the expression of DHHC5 was detected by WB. *, *P* < 0.05, compare to the control group or corresponding group. (For interpretation of the references to colour in this figure legend, the reader is referred to the web version of this article.)

Treatment with the palmitoylation inhibitor 2-BP revealed that palmitoylation intervention could inhibit mitochondrial quality control and metabolic reprogramming induced by S protein expression. Compared to S-expressing cells, 2-BP-treated S-expressing cells exhibited decreased levels of Drp1 and p-Drp1^Ser616^, along with an increase in Mfn2 levels (Fig. 5A and Fig. S5A). FCM analysis revealed a rescue in the fluorescence intensity of the mtSOX probe (Fig. 5B and Fig. S5B); in addition, mitochondrial membrane potential was repolarized in 2-BP-treated S-expressing cells (Fig. 5C and Fig. S5C). Moreover, the activity of hexokinase and pyruvate kinase, as well as lactic acid content, were lower in 2-BP-treated S-expressing cells (Fig. 5D and Fig. S5D). These findings suggest that palmitoylation can rescue the increased glycolytic activity in lung epithelial cells expressing the S protein. To elucidate whether palmitoylation can regulate the interaction between COX-2 and HK2, the Acyl-RAC assay was used to assess the palmitoylation level of COX-2. In the Acyl-RAC assay, the addition of HAM selectively cleaves the thioester bonds of palmitoylated proteins, allowing for the detection of palmitoylated COX-2, which is represented as COX-2 (Palm) in the results. Subsequently, mitochondrial fractions were isolated for Mito-IP analysis. Palmitoylation of COX-2 was observed in S-expressing cells (Fig. 5E and Fig. S5E). There was a reduction in both COX-2 palmitoylation and COX-2-HK2 complex formation in the mitochondrial fraction of 2-BP-treated S cells (Fig. 5F-G and Fig. S5F-G). Conversely, treatment with the depalmitoylation inhibitor ML348 resulted in increased COX-2 palmitoylation levels and COX-2-HK2 complex formation in the mitochondrial fractions (Fig. 5H-I and Fig. S5H-I). Collectively, these results suggest that SARS-CoV-2 S protein expression mediates mitochondrial metabolic reprogramming by regulating COX-2 palmitoylation and the COX-2-HK2 complex in the mitochondria of lung epithelial cells.

Next, we used GPS-Palm v2.0, a palmitoylation prediction algorithm, to analyze the palmitoylation sites on COX-2. Site-specific mutations with high prediction scores were introduced at five sites to generate COX-2 palmitoylation-deficient cell lines (Fig. 5J and Fig. S5J). Notably, the C555S mutation in COX-2 led to a significant reduction in palmitoylation induced by the S protein (Fig. 5K and Fig. S5K) and was associated with decreased levels of the mitochondrial COX-2-HK2 complex (Fig. 5L-M), indicating that S protein expression regulates mitochondrial reprogramming via palmitoylation at the COX-2^Cys555^ site. We also investigated the palmitoyltransferase responsible for COX-2 palmitoylation. Analysis of the GSE147507 database of SARS-CoV-2-infected A549 cell samples identified a significant correlation between *PTGS2* and the acyltransferase *DHHC5,* which is a candidate gene for experimental verification (*r* = 0.8857, *P* < 0.05) (Fig. 5N-O). *DHHC5* mRNA expression increased in S-expressing cells (Fig. 5P). To investigate the interaction between palmitoyltransferases and their substrates, COX-2 was overexpressed in A549-*PTGS2* cells using the pB-*PTGS2* plasmid. Immunoprecipitation revealed a physical interaction between FLAG-tagged COX-2 and DHHC5 (Fig. 5Q), which was hypothesized to underline DHHC5-mediated COX-2 palmitoylation. Stable Cas9-based *DHHC5* knockdown cells, A549-*DHHC5*-KD, were constructed (Fig. S5L). Compared to control cells expressing the S protein, A549-*DHHC5*-KD cells expressing the S protein exhibited decreased COX-2 palmitoylation and decreased COX-2-HK2 complex formation in the mitochondrial fraction (Fig. 5R-S). The C555S mutation in COX-2 also prevented physical interaction between DHHC5 and COX-2 (Fig. 5T). These findings suggest that SARS-CoV-2 S protein expression regulates COX-2^Cys555^ palmitoylation via DHHC5, thereby modulating mitochondrial metabolic reprogramming through the COX-2-HK2 complex in the mitochondria.

### SARS-CoV-2 S protein-induced mitochondrial metabolic reprogramming mediates pyroptosis in inflammatory lung tissue *in vivo*

**Fig. 6:**
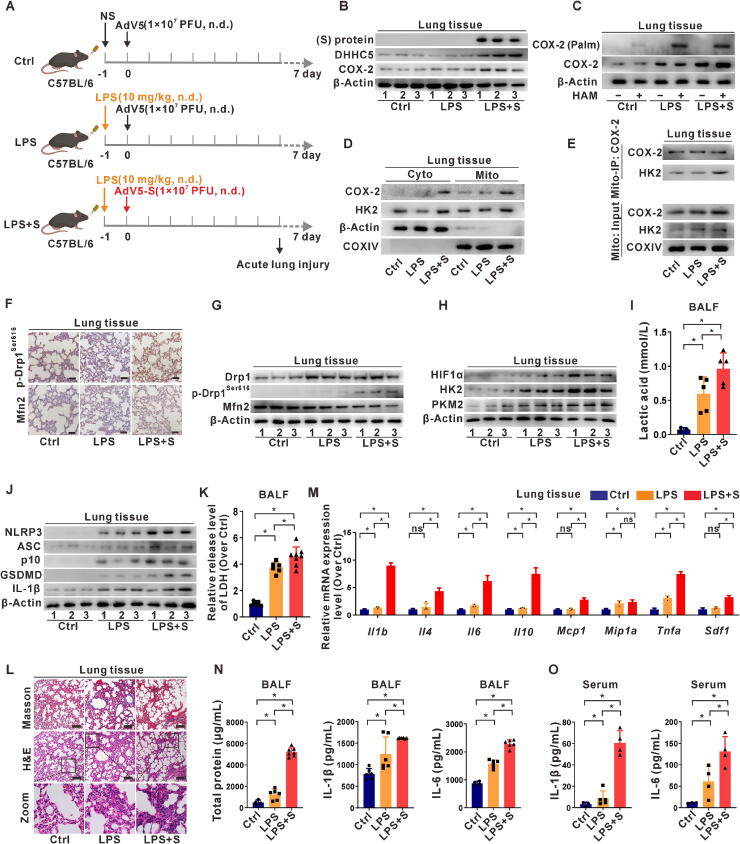
**SARS-CoV-2 S protein-induced mitochondrial metabolic reprogramming mediates pyroptosis in inflammatory lung tissue *in vivo.* (A)** An overview of the animal experiment (five mice per group). C57BL/6 mice received intranasal administration of LPS (10 mg/kg) or normal saline (NS), followed by intranasal injection of AdV5-S (1 × 10^7^ PFU) or AdV5-empty after 24 h. (**B)** WB analysis of SARS-CoV-2 S protein, DHHC5, and COX-2 proteins in lung tissues. (**C)** The level of palmitoylated COX-2 was detected by the Acyl-RAC assay. (**D)** WB analysis of COX-2 and HK2 expression in cytoplasmic (Cyto) and mitochondrial (Mito) fractions. (**E)** Mito-IP with an anti-COX-2 antibody, and the expression of HK2 was detected by WB. (**F)** Immunohistochemistry (IHC) staining was used to evaluate p-Drp1^Ser616^ and Mfn2 in paired lung tissues from mice. Scale bar, 50 μm. (**G)** WB analysis of mitochondrial dynamics proteins (Drp1, p-Drp1^Ser616^, and Mfn2). (**H)** WB analysis of glycolysis-related proteins (HIF1α, HK2, and PKM2). (**I)** Levels of lactic acid in BALF. (**J)** WB analysis of NLRP3 inflammasome activation (NLRP3, ASC, and p10) and pyroptosis-related proteins (GSDMD and IL-1β). (**K)** LDH release assay in BALF. (**L)** Masson's and H&E staining were used to evaluate lung pathological changes. Scale bar, 50 μm. (**M)** mRNA levels of inflammation-related genes in lung tissues were measured by qRT-PCR. (**N)** Levels of total protein and inflammatory cytokines (IL-1β and IL-6) in BALF. (**O)** ELISA analysis of inflammatory cytokines (IL-1β and IL-6) levels in serum. *, *P* < 0.05, compare to the control group or corresponding group.

To further investigate the mechanism underlying pneumonia-induced injury caused by SARS-CoV-2 S protein expression, a mouse model of S protein expression was constructed using the adenoviral vector AdV5. As S protein expression alone induced only minor histopathological changes in mice, an LPS-induced acute lung injury model was established to exacerbate lung injury via nasal administration of AdV5-S (Fig. 6A). S protein expression and levels of DHHC5 and COX-2 in lung tissues increased, confirming the successful construction of the mouse model with S protein expression (Fig. 6B). Compared to the NS- or LPS-treated groups, an increase in COX-2 palmitoylation was observed in lung tissues from the LPS + S group (Fig. 6C). Furthermore, mitochondrial distribution and COX-2 and HK2 complex formation increased in this group (Fig. 6D-E), indicating that S protein expression promotes the interaction of mitochondrial COX-2 with HK2 by inducing COX-2 palmitoylation *in vivo*. IHC and western blotting revealed increased levels of p-Drp1^Ser616^ and decreased levels of Mfn2 in the lung tissues from mice in the LPS + S group compared to those treated with NS or LPS alone (Fig. 6F-G). Glycolysis levels were also elevated in the lung tissues of mice in the LPS + S protein expression group, as indicated by increased expression levels of glycolytic rate-limiting enzymes and higher lactic acid levels in the BALF (Fig. 6H-I). These findings suggest that S protein expression in infected lung tissues can increase glycolysis levels. Compared to the control group, the levels of NLRP3 inflammasome and pyroptosis-related proteins in lung homogenates increased in mice treated with LPS and were further enhanced in the LPS + S-expressing group (Fig. 6J). LDH release in the BALF also increased (Fig. 6K), indicating that S protein expression can induce NLRP3 inflammasome activation and pyroptosis in mice lung tissues. Histological changes in lung tissues were observed using Masson's and H&E staining. Compared to the control group, LPS-treated mice exhibited increased deposition of elastic collagen around the bronchioles, destruction of alveolar structure, decreased compensatory capacity of alveolar cells, and increased infiltration of alveolar interstitial inflammatory cells (Fig. 6L).

Additionally, qRT-PCR analysis of lung tissues revealed upregulation of cytokine and chemokine transcripts, including *Il1b*, *Il4*, *Il6*, *Il10*, *Mcp1*, and *Sdf1*, in the LPS + S-expressing group compared to those in mice treated with NS or LPS alone (Fig. 6M). Furthermore, compared to the control group, the protein concentration in BALF increased significantly (*P* < 0.05), indicating fluid imbalance in the lungs, characterized by leakage of a large amount of proteins from pulmonary microvessels and the occurrence of inflammatory reactions. ELISA results showed increased levels of IL-1β and IL-6 in the BALF and serum of LPS + S-expressing mice compared to those treated with LPS alone or the control group (Fig. 6N-O). In summary, the results indicate that SARS-CoV-2 S protein expression can activate the NLRP3 inflammasome in lung tissue, mediating pyroptosis and promoting acute inflammatory lung injury.

### Role of DHHC5 knockdown in regulating SARS-CoV-2 S protein-induced mitochondrial COX-2 palmitoylation-mediated pyroptosis in lung tissues *in vivo*

**Fig. 7:**
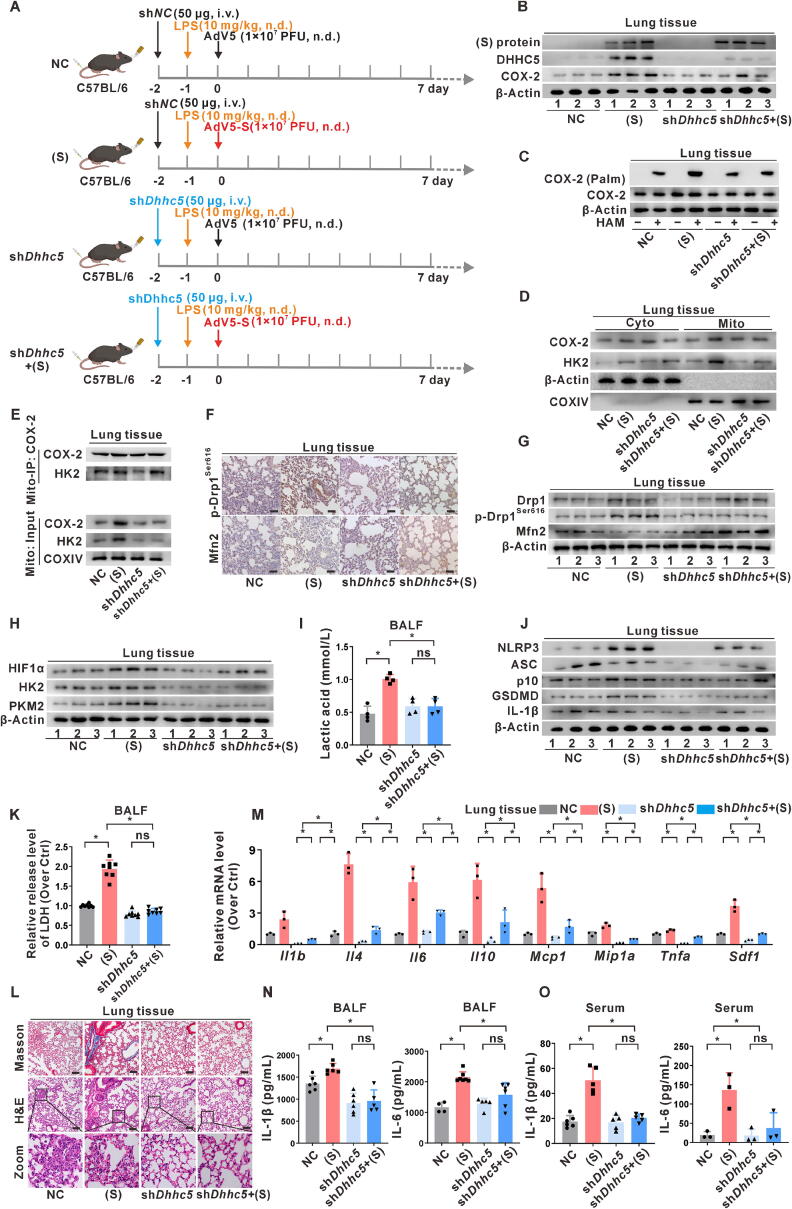
**Role of DHHC5 knockdown in regulating SARS-CoV-2 S protein-induced mitochondrial COX-2 palmitoylation-mediated pyroptosis in lung tissues *in vivo.* (A)** An overview of the animal experiment (five mice per group). C57BL/6 mice were given sh*Dhhc5* intervention (50 μg, i.v.), followed by intranasal administration of LPS (10 mg/kg) or normal saline (NS) after 24 h, and then intranasal injection of AdV5-S or AdV5-empty (1 × 10^7^ PFU). (**B)** WB analysis of SARS-CoV-2 S protein, DHHC5, and COX-2 proteins in lung tissues. (**C)** The level of palmitoylated COX-2 was detected by the Acyl-RAC assay. (**D)** WB analysis of COX-2 and HK2 expression in cytoplasmic (Cyto) or mitochondrial (Mito) fractions. (**E)** Mito-IP with an anti-COX-2 antibody, and the expression of HK2 was detected by WB. (**F)** IHC staining was used to evaluate p-Drp1^Ser616^ and Mfn2 in paired lung tissues from mice. Scale bar, 50 μm. (**G)** WB analysis of mitochondrial dynamics proteins (Drp1, p-Drp1^Ser616^, and Mfn2). (**H)** WB analysis of glycolysis-related proteins (HIF1α, HK2, and PKM2). (**I)** Detection of lactic acid levels in BALF. (**J)** WB analysis of NLRP3 inflammasome activation (NLRP3, ASC, and p10) and pyroptosis-related proteins (GSDMD and IL-1β). (**K)** LDH release assay in BALF. (**L)** Masson's and H&E staining were used to evaluate lung pathological changes. Scale bar, 50 μm. (**M)** mRNA levels of inflammation-related genes in lung tissues were measured by qRT-PCR. (**N-O)** Levels of inflammatory factors (IL-1β and IL-6) in BALF (**N**) and serum (O). *, *P* < 0.05, compare to the control group or corresponding group.

To achieve DHHC5 knockdown in mice, DOTAP:Chol liposomes encapsulating the plasmid sh*Dhhc5* were used for intravenous injection via the tail vein. This intravenous administration of plasmid DNA/DOTAP liposomes resulted in high gene expression efficiency in lung tissue. As shown in Fig. 7A, a *Dhhc5* knockdown model was successfully constructed, followed by the induction of acute inflammatory lung injury in mice via S protein expression. Western blotting revealed decreased levels of both DHHC5 and COX-2 in the lung tissues of mice in the *Dhhc5*-targeted intervention S-expressing group (Fig. 7B). These findings confirmed the successful knockdown of *Dhhc5* in the lungs of S-expressing mice. Furthermore, compared to the S-expressing group, a reduction in COX-2 palmitoylation levels was observed in the lung tissues of mice in the *Dhhc5-*targeted intervention S-expressing group (Fig. 7C), indicating that DHHC5 plays a regulatory role in COX-2 palmitoylation induced by S protein expression. To investigate suborganellar distribution, mitochondrial fractions were extracted from mouse lung tissues to assess the distribution of COX-2 and HK2. The mitochondrial distribution and mitochondrial complex formation of COX-2 and HK2 decreased in the lung tissues of mice in the S-expressing group, and were further reduced in the lung tissues of mice in the *Dhhc5-*targeted intervention S-expressing group (Fig. 7D-E), suggesting that DHHC5-mediated palmitoylation of COX-2 is involved in regulating mitochondrial distribution in the S protein expression model *in vivo*. Moreover, IHC staining and WB analysis revealed a reduction in p-Drp1^Ser616^ levels in the lung tissues of mice in the *Dhhc5*-targeted intervention S-expressing group compared to those in the S-expressing group (Fig. 7F-G). Glycolysis levels were notably reduced in the lung tissues of mice in the *Dhhc5*-targeted intervention S-expressing group compared to those in the S-expressing group, as indicated by the downregulated expression levels of glycolytic rate-limiting enzymes in lung tissues and decreased levels of lactic acid in the BALF (Fig. 7H-I).

Additionally, the levels of NLRP3 inflammasome and pyroptosis-related proteins in the lung tissues of the *Dhhc5*-targeted intervention S-expressing group were lower than those in the S-expressing group (Fig. 7J). Moreover, there was a reduction in LDH release in the BALF (Fig. 7K), indicating that *Dhhc5*-targeted intervention inhibited NLRP3 inflammasome activation and pyroptosis in the lung tissues of mice induced by S protein expression. Histopathological changes in the lung tissues were assessed using Masson's and H&E staining. As shown in Fig. 7L, mouse lungs in the *Dhhc5*-targeted intervention S-expressing group exhibited reduced elastic collagen deposition around the bronchioles, restored alveolar structure, and decreased inflammatory cell infiltration in the alveolar interstitium compared to those in the S-expressing group. These findings suggest that S protein expression contributes to increased lung inflammation in mice, mediated by DHHC5 induction. Furthermore, qRT-PCR analysis revealed reduced transcription levels of cytokines and chemokines in lung tissues (*P* < 0.05) (Fig. 7M). ELISA showed decreased levels of IL-1β and IL-6 in the BALF and serum of the *Dhhc5*-targeted intervention S-expressing group compared to the S-expressing group (Fig. 7N-O). Collectively, these findings suggest that DHHC5 knockdown reduces the hyperinflammatory status in the lungs of mice by inhibiting S protein-induced COX-2 palmitoylation and its mitochondrial distribution, as well as by inhibiting NLRP3 inflammasome activation and pyroptosis.

### Interventive effects of DHHC5 on prolonged SARS-CoV-2 S protein-induced inflammatory neuro-olfactory PACS

**Fig. 8:**
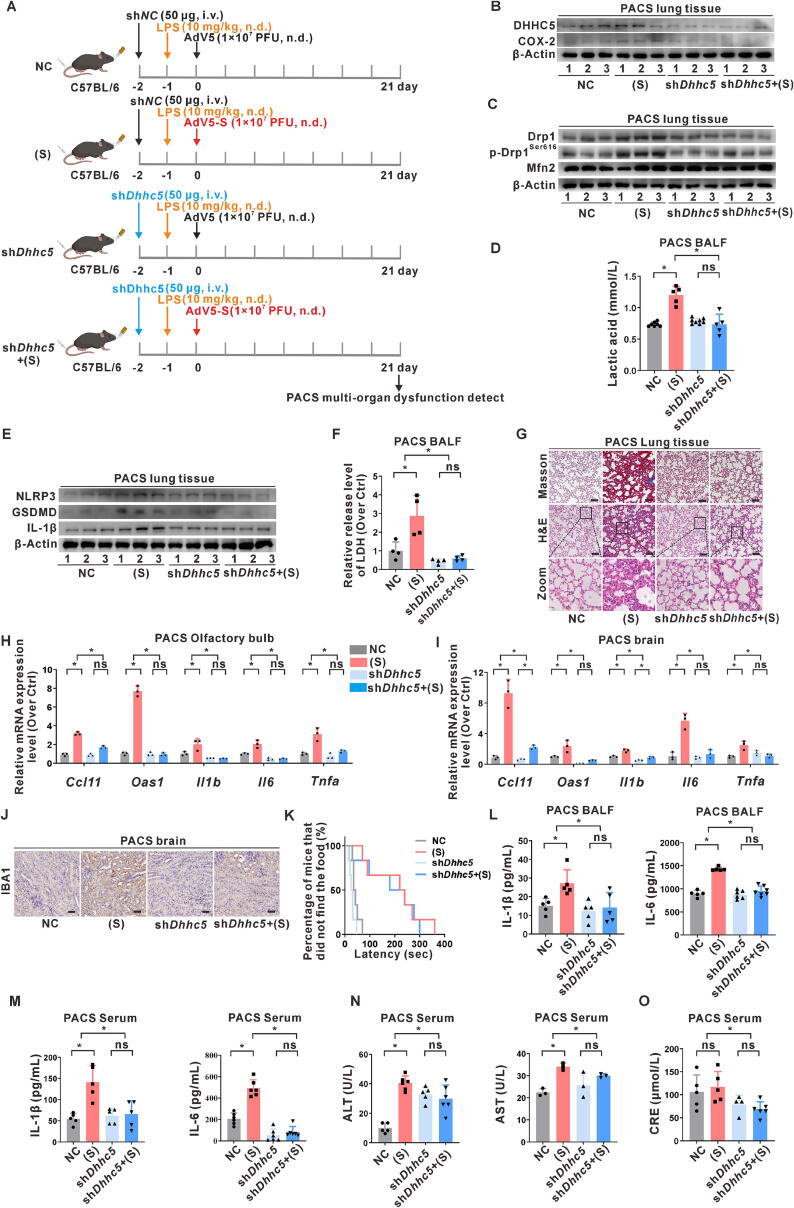
**Interventive effects of DHHC5 on prolonged SARS-CoV-2 S protein-induced inflammatory neuro-olfactory PACS. (A)** An overview of the animal experiment (five mice per group). C57BL/6 mice were given sh*Dhhc5* intervention (50 μg, i.v.), followed by intranasal administration of LPS (10 mg/kg) or NS after 24 h, and then intranasal injection of AdV5-S (1 × 10^7^ PFU) or AdV5-empty. Mice were sacrificed 21 d after administration to construct a PACS model. (**B)** WB analysis of DHHC5 and COX-2 proteins in lung tissues. (**C)** WB analysis of mitochondrial dynamics proteins (Drp1, p-Drp1^Ser616^, and Mfn2). (**D)** Detection of lactic acid levels in BALF. (**E)** WB analysis of NLRP3 inflammasome activation (NLRP3, ASC, and p10) and pyroptosis-related proteins (GSDMD and IL-1β). (**F)** LDH release assay in BALF. (**G)** Masson's and H&E staining were used to evaluate lung pathological changes. Scale bar, 50 μm. (**H-I)** mRNA levels of *Ccl11*, *Oas1*, *Il6*, *Il1b*, and *Tnfa* in the olfactory bulb **(H)** and brain **(I)** were measured by qRT-PCR. (**J)** IHC staining of IBA1 in the brain. (**K)** The percentage of mice finding food over time, with impaired sense of smell detected. **(L)** Levels of inflammatory factors (IL-1β and IL-6) in BALF. (**M)** Levels of inflammatory factors (IL-1β and IL-6) in serum. (**N-O)** Detection of ALT, AST **(N),** and creatinine **(O)** in serum. *, *P* < 0.05, compare to the control group or corresponding group.

To investigate the involvement of prolonged S protein expression in PACS-related multiple organ damage, the exposure period was extended to 21 days (Fig. 8A). Western blotting analysis revealed decreased levels of DHHC5 and COX-2 proteins in lung tissues of mice in the *Dhhc5*-targeted intervention S-expressing group compared to those in the S-expressing group (Fig. 8B), indicating that S protein expression can induce long-term lung disruption in mice, which can be mitigated by sh*Dhhc5* intervention. Western blotting analysis also revealed decreased levels of p-Drp1^Ser616^ in lung tissues of mice in the *Dhhc5*-targeted intervention S-expressing group compared to those in the S-expressing PACS group (Fig. 8C). Glycolysis levels were reduced in PACS lung tissues, as evidenced by decreased lactic acid levels in the BALF (Fig. 8D). Furthermore, the activation of the NLRP3 inflammasome and pyroptosis in lung tissues was evaluated. Compared to the S-expressing group, the levels of NLRP3 inflammasome and pyroptosis-related proteins in lung tissues of the *Dhhc5*-targeted intervention S-expressing group were decreased (Fig. 8E), along with reduced LDH release in the BALF (Fig. 8F). These findings indicate that S protein expression can induce persistent lung inflammation associated with pyroptosis, while targeted intervention of DHHC5 can rescue NLRP3 inflammasome activation and pyroptosis in lung tissues of mice induced by S protein expression. Histopathological changes in lung tissues were assessed using Masson's and H&E staining. In the S-expressing group, significant elastic collagen deposition and inflammatory cell infiltration were observed in alveolar spaces. In contrast, the alveolar structure was largely restored by DHHC5 intervention (Fig. 8G), indicating that S protein expression induces inflammatory injury in mice lung tissues. Simultaneously, DHHC5 intervention alleviated the pathological damage induced by S protein expression.

Considering previous reports of long-term cognitive dysfunction in mice resembling PACS [40], the olfactory bulbs and brain tissues of mice with PACS were processed for further analysis. S protein expression was found to significantly induce neuro-olfactory PACS symptoms, as evidenced by increased transcription levels of nerve injury and cognitive impairment marker (*Ccl11*), PACS marker (*Oas1*), and pro-inflammatory cytokine genes (*Il6*, *Il1b*, and *Tnfa*) in both the olfactory bulb and brain (Fig. 8H-I). IHC demonstrated an increase in the level of the microglial marker IBA1 in brain tissue (Fig. 8J), and the mice exhibited olfactory dysfunction, characterized by an impaired sense of smell (Fig. 8K). Early intervention targeting DHHC5 could suppress the presentation of neuro-olfactory PACS in mice, suggesting that S protein expression induces neuro-olfactory PACS, which can be mitigated by DHHC5-targeted intervention. The level of IL-1β in BALF was significantly lower, while the level of IL-6 remained higher in the DHHC5-intervened group than in the acute inflammatory stage (Fig. 8L). Similarly, the levels of pro-inflammatory cytokines in the sera were higher than those in the acute stage but were inhibited by DHHC5 intervention (Fig. 8M), suggesting a shift toward chronic persistent inflammation associated with PACS in mice post-acute pneumonic injury. Furthermore, S protein expression led to a slight increase in liver and kidney function markers (alanine aminotransferase, aspartate aminotransferase, and creatinine), which remained within normal ranges (Fig. 8N-O). In summary, these findings suggest the successful establishment of a neuro-olfactory PACS mouse model induced by S protein expression and highlight DHHC5 as a potential intervention target for PACS.

## Discussion

Recent advancements in the understanding of protein palmitoylation have highlighted its significance in disease pathogenesis and its potential as a therapeutic target. The importance of palmitoylation is widely recognized, particularly in inflammatory and carcinogenic diseases. For example, the effects of DHHC20 antagonists on EGFR inhibition in KRAS mutant tumors illustrate its key role in cancer biology [41]. In the context of viral infections, protein palmitoylation acts as a dynamic regulator of viral replication. A recent study emphasized the critical role of palmitoylation in coronavirus replication and the formation of viral membrane structures, suggesting promising therapeutic avenues, especially during the COVID-19 pandemic [42]. COVID-19, caused by SARS-CoV-2, is characterized by a cytokine storm and an exaggerated inflammatory response. Although vaccination efforts have successfully reduced the risk of SARS-CoV-2 infection and severe illness, long-term consequences, known as PACS, continue to affect patient well-being [24]. Long COVID is defined by chronic inflammation and immune dysregulation. Increased glycolysis and altered mitochondrial function are vital aspects of worsening metabolic dysfunction during and after the pandemic, and they are strongly linked to various systemic illnesses [43]. Long COVID manifests as a variety of symptoms that affect multiple organ systems, including fatigue, cognitive dysfunction, and respiratory disorders. The potential link between mild to moderate COVID-19 symptoms and an exaggerated inflammatory response has sparked interest in the use of anti-inflammatory drugs, such as CELE, as a preventive treatment in the early stages of the disease [44]. A comprehensive understanding of the intricate relationship between COX-2 and lung inflammatory injury is pivotal for unraveling the underlying mechanisms driving the pathogenesis of COVID-19. The loss of cell adhesion proteins in lung epithelial cells and disruption of mitochondrial quality control compromise the integrity of the lung epithelial barrier, leading to chronic damage. These findings suggest that alterations in mitochondrial metabolism represent a critical pathogenic mechanism associated with the S protein expression during SARS-CoV-2 infection [45]. Additionally, research has revealed that the SARS-CoV-2 S protein is a major mediator of neuroinflammation, cognitive impairment, and nervous system abnormalities in animal models [40]. These findings are particularly relevant to PACS, as both inflammatory and tolerogenic roles of the SARS-CoV-2 S protein have been demonstrated, particularly in the presence of LPS. Immune tolerance may account for the prolonged presence of the S protein in the body, which can persist for months. Repeated exposure to the same antigen, whether through infections or COVID-19 vaccinations, may result in cumulative deleterious effects, potentially leading to amyloid formation and neuroinflammatory activities [46].

Our study elucidated the significance of COX-2 palmitoylation in promoting lung epithelial cell damage and inflammation. Pharmacological inhibition of COX-2 palmitoylation leads to a reduction in the formation of the COX-2-HK2 mitochondrial complex. This intervention also results in decreased mitochondrial metabolic reprogramming and reduced activation of the NLRP3 inflammasome triggered by S protein expression. Palmitoylated COX-2 significantly impacts mitochondrial function and energy metabolism, disrupts cellular processes, and exacerbates inflammatory lung injury via mitochondrial transport. Furthermore, treatment with CELE effectively suppresses mitochondrial metabolic reprogramming and NLRP3 inflammasome activation induced by S protein expression *in vitro*. *In vivo* studies have demonstrated that inhibiting palmitoylated COX-2 rescues mice from COVID-19 pneumonia, underscoring the potential of targeting the NLRP3 inflammasome pathway via COX-2 modulation as a therapeutic strategy. The implications for long COVID are profound, as persistent inflammation and mitochondrial dysfunction may underlie many of the chronic injuries and symptoms experienced by patients.

By extending the exposure time to 21 days, we investigated the role of the S protein in the damage to multiple organs associated with PACS. We found that S protein expression induced subacute inflammatory injury, persistent metabolic alterations, and pyroptosis in lung tissues, leading to chronic inflammation typical of PACS *in vivo*. Importantly, our study demonstrated that targeted intervention with DHHC5, an enzyme involved in protein palmitoylation, could mitigate the effects of S protein expression-induced organ inflammation and damage. This study also explored the DHHC5-COX-2-NLRP3 signaling cascade as a potential molecular pathway for SARS-CoV-2-induced lung epithelial cytotoxicity and pneumonia. Genetic intervention with *Dhhc5* inhibited neuro-olfactory dysfunction associated with PACS induced by S protein expression, thereby reducing cognitive impairment and olfactory dysfunction in mice. Thus, DHHC5 may serve as a promising therapeutic target for treating COVID-19 and mitigating PACS-related complications.

In summary, our findings highlight the crucial role of mitochondrial COX-2 in lung cell lines expressing the SARS-CoV-2 S protein and its relevance to long COVID. The interaction between chronic inflammation and mitochondrial dysfunction is pivotal, as these processes are central to the pathophysiology of long COVID, characterized by symptoms such as cognitive impairment and respiratory difficulties that persist long after acute infection. Furthermore, the identification of DHHC5 as a therapeutic target enhances our understanding of long COVID, highlighting the dual role of the SARS-CoV-2 S protein in inflammation and immune tolerance. This complexity reflects the intricacies of the immune response and the potential for cumulative tissue damage over time, which is further exacerbated by the risk of reactivating of latent viral infections. These insights may inform innovative strategies for managing long COVID and other post-viral syndromes, advancing our understanding of the role of protein palmitoylation and enabling more efficient preventive and therapeutic approaches for infected individuals and those with chronic diseases.

## Limitations of this study

First, although our study suggests that COX-2 palmitoylation is crucial in SARS-CoV-2 S protein expression-induced lung inflammation and injury, targeting this modification may also alleviate multi-organ damage in PACS. However, we lack dynamic observations in both *in vitro* and *in vivo* models to better elucidate the role of mitochondrial COX-2 in ALI and PACS. Second, our mouse model lacks other viral components, such as membrane proteins or viral single-stranded RNA, which may limit its ability to fully replicate the complexity of human SARS-CoV-2 infection and long COVID-19 manifestations. Finally, our study focused on lung epithelial cells and did not investigate the role of intercellular communication with other cell types, such as macrophages [47]. Addressing these limitations is critical for developing effective clinical strategies for treating COVID-19 and its long-term complications.

## Compliance with ethics requirements

*All animal experiments were conducted in accordance with the guidelines of Xiamen University Animal Ethical Committee. The animal experiments protocols were approved by the Ethical Committee for Animal Experimentation at Xiamen University (Ethical Approval Number: XMULAC20200201, dated 2020-02-01).*

## CRediT authorship contribution statement

**Jia-Shen Wu:** Investigation, Methodology, Writing – original draft, Writing – review & editing. **Chi-Yu Xu:** Methodology, Investigation, Data curation, Writing – original draft. **Su-Min Mo:** Methodology, Data curation. **Xin-Mou Wu:** Methodology. **Ze-Bang Du:** Conceptualization. **Lin Che:** Conceptualization. **Yi-Ling Zhang:** Data Curation. **Kai-Li Yang:** Methodology. **Ting-Dong Li:** Resources. **Sheng-Xiang Ge:** Conceptualization, Resources. **Tian-Ying Zhang:** Resources. **Zhong-Ning Lin:** Writing – review & editing, Funding acquisition. **Yu-Chun Lin:** Supervision, Writing – review & editing.

## Funding

This work was supported by the 10.13039/501100001809National Natural Science Foundation of China (Nos. 82273667, 82073588, 81973082), The Industry-University-Research Cooperation Project of Fujian Science and Technology Plan (No. 2022Y4009), XMU Undergraduate Innovation and Entrepreneurship Training Programs (Nos. S202010384747, 2019Y0810, 2019X0797), and Innovative Practice Platform for Undergraduate Students, School of Public Health Xiamen University (No. 202005).

## Declaration of competing interest

*The authors declare that they have no known competing financial interests or personal relationships that could have appeared to influence the work reported in this paper.*
