# Supplementary material for: Palmitoylated COX-2Cys555 reprogrammed mitochondrial metabolism in pyroptotic inflammatory injury in patients with post-acute COVID-19 syndrome
Source: J Adv Res. 2025 May 9;80:331–49. doi: 10.1016/j.jare.2025.05.005 (PMC12869196; doi:10.1016/j.jare.2025.05.005)
Supplement: Supplementary Data 1 [file mmc1.docx]

Supplementary materials

**Palmitoylated COX-2^Cys555^ reprogramed mitochondrial metabolism in SARS-CoV-2 Spike protein-induced NLRP3-dependent lung inflammatory damage in post-acute COVID-19 syndrome**

Jia-Shen Wu^#^, Chi-Yu Xu^#^, Su-Min Mo^#^, Xin-Mou Wu, Ze-Bang Du, Lin Che, Yi-Ling Zhang, Kai-Li Yang, Ting-Dong Li, Sheng-Xiang Ge, Tian-Ying Zhang, Zhong-Ning Lin*, Yu-Chun Lin*

State Key Laboratory of Vaccines for Infectious Diseases, Xiang An Biomedicine Laboratory, Xiang'an Hospital of Xiamen University, National Innovation Platform for Industry-Education Integration in Vaccine Research, School of Public Health, Xiamen University, Xiamen 361102, China.

# These authors contributed equally to this work.

* Corresponding Author: State Key Laboratory of Vaccines for Infectious Diseases, Xiang An Biomedicine Laboratory, Xiang'an Hospital of Xiamen University, National Innovation Platform for Industry-Education Integration in Vaccine Research, School of Public Health, Xiamen University, Xiang′an South Road, Xiamen, 361102, China.

Tel: +86 592 2880615; Fax: +86 592 2881578

E-mail: [linzhn@xmu.edu.cn](mailto:linzhn@xmu.edu.cn) (Zhong-Ning Lin), [linych@xmu.edu.cn](mailto:linych@xmu.edu.cn) (Yu-Chun Lin)

**1. Supplementary tables**

**Table S1. Primer sequences used in the present study**

| Primers | Sequence (5'-3') |
| --- | --- |
| sh*Dhhc5* FP | CCGGCGCACAACCAATGAACAGGTTCTCGAGAACCTGTTCATTGGTTGTGCGTTTTTG |
| sh*Dhhc5* RP | AATTCAAAAACGCACAACCAATGAACAGGTTCTCGAGAACCTGTTCATTGGTTGTGCG |
| *DHHC5*-sgRNA-1 FP | CACCGCACTGCAGGTGACACATAC |
| *DHHC5*-sgRNA-1 RP | AAACGTATGTGTCACCTGCAGTGC |
| *DHHC5*-sgRNA-2 FP | CACCGCCAGACCTGAGCCGTTACAC |
| *DHHC5*-sgRNA-2 RP | AAACGTGTAACGGCTCAGGTCTGGC |
| *ND4L* FP | CACCCACTCCCTCTTAGCC |
| *ND4L* RP | TAGCATTGGAGTAGGTTTAGGTT |
| *NDUFA4* FP | CCCCTCTTTGTATTTATTGGAAC |
| *NDUFA4* RP | GGCTCTGGGTTATTTCTGTCC |
| *ATP6* FP | ACACAACACTAAAGGACGAACC |
| *ATP6* RP | GTGTAAATGAGTGAGGCAGGAG |
| *ATP8* FP | CTACCACCTACCTCCCTCACC |
| *ATP8* RP | GGGCAATGAATGAAGCGAAC |
| *SLC2A1* FP | GTGGGCATGTGCTTCCAGTAT |
| *SLC2A1* RP | CAGCTCCTCGGGTGTCTTGT |
| *HK2* FP | TTCTTGGCCTTGGACCTTG |
| *HK2* RP | CCAGATGCCTTGAAGCCTTTT |
| *PKM2* FP | TCCTCCTGAAGGTGACTGC |
| *PKM2* RP | TGGGTGGTGAATCAATGTCC |
| *LDHA* FP | CCAAGCTGGTCATTATCACGG |
| *LDHA* RP | CATTCCACTCCATACAGGCAC |
| *CPT1A* FP | ATTTCCATTCCTTCCCATTCG |
| *CPT1A* RP | ACTTCAGCCTCTGTTCCACC |
| *PPARA* FP | CCCTCCTCGGTGACTTATCC |
| *PPARA* RP | ACTGGCATTTGTTTCTGTTCTTT |
| *CYP3A4* FP | GCAGTTTTACCCAATAAGGC |
| *CYP3A4* RP | ATCTTTCAGGGAGGAACTTC |
| *PTGS2* FP | ATGCTCGCCCGCGCCCT |
| *PTGS2* RP | CTACAGTTCAGTCGAACGTTCT |
| *DHHC5* FP | GAAGACTGAAGAAAGATAAGAGACATTG |
| *DHHC5* RP | GACACTTCAAAAGTTTACTGTGGATG |
| *IL1B* FP | CAGTGGCAATGAGGATGACTTG |
| *IL1B* RP | GCTGTAGTGGTGGTCGGAGATT |
| *IL6* FP | CTTCGGTCCAGTTGCCTTCT |
| *IL6* RP | CCAGTGCCTCTTTGCTGCTTT |
| *IL18* FP | TCAAGACCAGCCTGACCAAC |
| *IL18* RP | CTCACCACAACCTCTACCTCC |
| *NFKB1* FP | GAAATTCCTGATCCAGACAAAAAC |
| *NFKB1* RP | ATCACTTCAATGGCCTCTGTGTAG |
| *STAT3* FP | GAAATTCCTGATCCAGACAAAAAC |
| *STAT3* RP | ATCACTTCAATGGCCTCTGTGTAG |
| *NLRP1* FP | CACCATGGTAGTCCTGT |
| *NLRP1* RP | ACAGCCAGCCAACCG |
| *NRLP3* FP | CCATTCCCTGACCAGACTCTATG |
| *NRLP3* RP | GCAGGTAAAGGTGCGTGAGAT |
| *NLRC4* FP | GATGCTATAAAACTAGCTG |
| *NLRC4 R*P | TGTGAAGATTCTGAGCTAG |
| *AIM2* FP | GTTTGTAGTCCAGAAGGT |
| *AIM2* RP | GGCCTTAATAACCTTTATGG |
| *Il1b* FP | GCAACTGTTCCTGAACTCAACT |
| *Il1b* RP | ATCTTTTGGGGTCCGTCAACT |
| *Il4* FP | GGTCTCAACCCCCAGCTAGT |
| *Il4* RP | GCCGATGATCTCTCTCAAGTGAT |
| *Il6* FP | TAGTCCTTCCTACCCCAATTTCC |
| *Il6* RP | TTGGTCCTTAGCCACTCCTTC |
| *Il10* FP | GCTCTTACTGACTGGCATGAG |
| *Il10* RP | CGCAGCTCTAGGAGCATGTG |
| *Mcp1* FP | GCATTAGCTTCAGATTTACGGGT |
| *Mcp1* RP | TTAAAAACCTGGATCGGAACCAA |
| *Mip1a* FP | GCATTAGCTTCAGATTTACGGGT |
| *Mip1a* RP | TTAAAAACCTGGATCGGAACCAA |
| *Tnfa* FP | CCCTCACACTCAGATCATCTTCT |
| *Tnfa* RP | GCTACGACGTGGGCTACAG |
| *Sdf1* FP | TTCTTCAGCCGTGCAACAATC |
| *Sdf1* RP | TGCATCAGTGACGGTAAACCA |
| *Ccl11* FP | AGGCTCCATCCCAACTTCCTGCTG |
| *Ccl11* RP | AGACTATGGCTTTCAGGGTGCATC |
| *Oas1* FP | GGATCATCTTAACATCCTCAAG |
| *Oas1* RP | CACAGTTGGTACCAGTGCG |

**Table S2. Information on the primary antibodies used in the present study**

| Primary Antibodies | Manufacturers | Code Number | Dilution/Concentration | | | Molecule weight | Species |
| --- | --- | --- | --- | --- | --- | --- | --- |
|  |  |  | WB | IF | IHC |  |  |
| AIM2 | ABclonal | A3356 | 1:1000 |  |  | 40 KD | Rabbit |
| ASC | Santa Cruz | sc-514414 | 1:100 |  |  | 22KD | Mouse |
| β-Actin | Abclonal | AC026 | 1:5000 |  |  | 42kD | Rabbit |
| Bax | Beyotime | AF0057 | 1:1000 |  |  | 21KD | Rabbit |
| Bcl2 | Abclonal | A18415 | 1:1000 |  |  | 26KD | Rabbit |
| COX-2 | ABclonal | A1253 | 1:1000 | 1:100 | 1:100 | 68KD | Rabbit |
| COXⅣ | ABclonal | A11631 | 1:5000 |  |  | 20KD | Rabbit |
| Cyto C | Beyotime | AF1006 | 1:1000 |  |  | 40KD | Rabbit |
| DHHC5 | ABclonal | A18114 | 1:1000 |  |  | 78KD | Rabbit |
| Drp1 | ABclonal | A2705 | 1:1000 |  |  | 62KD | Rabbit |
| Flag | Beyotime | AF2852 | 1:1000 |  |  | 84KD | Mouse |
| GAPDH | ABclonal | AC002 | 1:5000 |  |  | 36KD | Mouse |
| GSDMD | Proteintech | 20770-1-AP | 1:2000 | 1:50 |  | 53KD | Rabbit |
| HIF1α | ABclonal | A7684 | 1:1000 |  |  | 93KD | Rabbit |
| HK2 | ABclonal | A0994 | 1:1000 |  |  | 102KD | Rabbit |
| IBA1 | Proteintech | 81728-1-RR | 1:10000 |  | 1:1000 | 17 kD | Rabbit |
| IL-1β | Abclonal | A16288 | 1:1000 |  |  | 31kD | Rabbit |
| Mfn2 | ABclonal | A19678 | 1:1000 |  | 1:100 | 86kD | Rabbit |
| NLRC4 | ABclonal | A7382 | 1:1000 |  |  | 116kD | Rabbit |
| NLRP3 | Santa Cruz | sc-134306 | 1:1000 |  |  | 106kD | Mouse |
| p10 | ABclonal | A23511 | 1:1000 |  |  | 11kD | Rabbit |
| p-Drp1Ser616 | Beyotime | AF5791 | 1:1000 |  | 1:100 | 80kD | Rabbit |
| PKM2 | ABclonal | A20991 | 1:1000 |  |  | 58kD | Rabbit |
| SARS-CoV-2  S protein | Gene Tax | GTX635654 | 1:1000 |  |  | 170kD | Rabbit |

**2. Supplementary figures and legends**

**
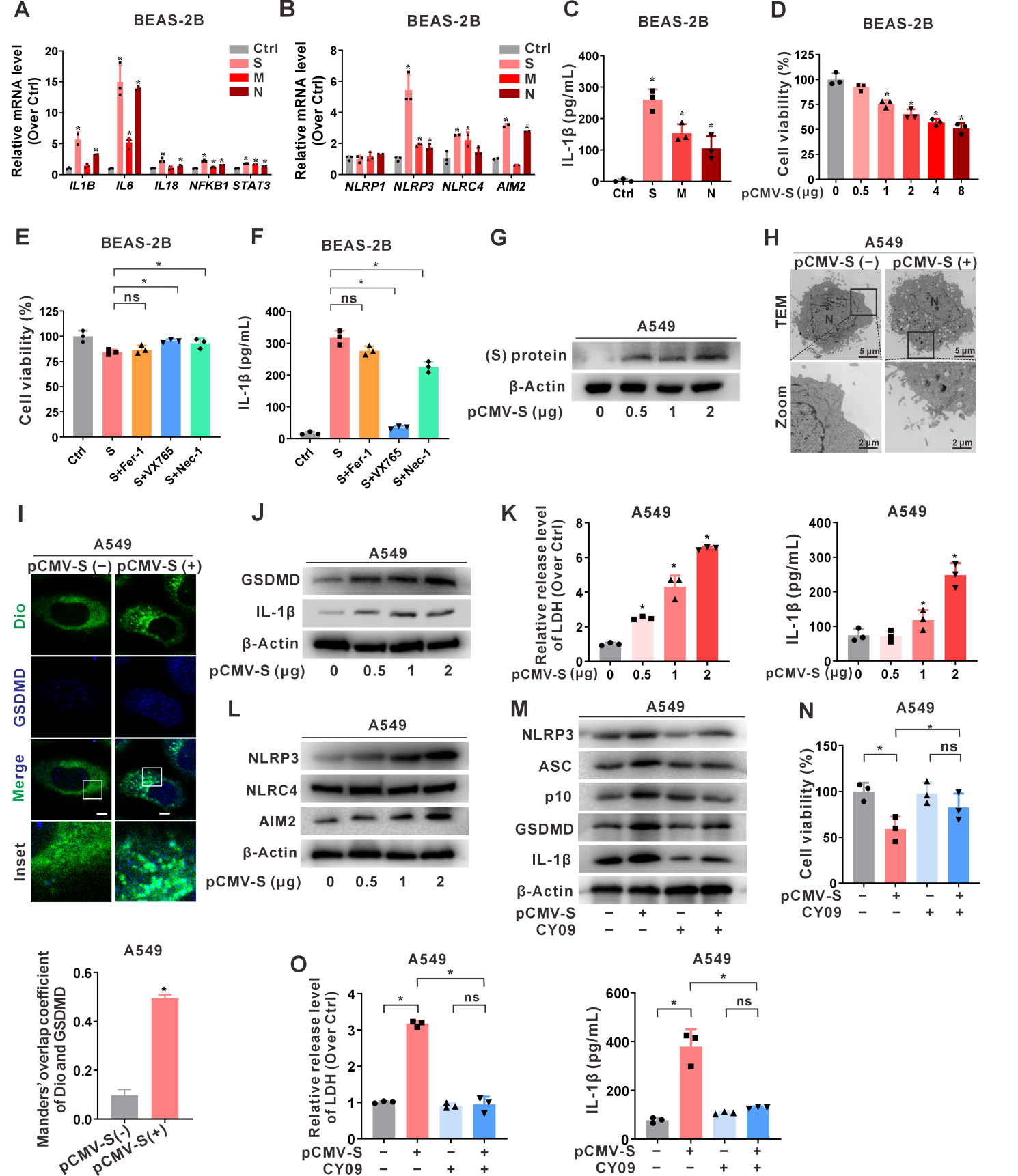
**

**Fig. S1. SARS-CoV-2 S protein-induced inflammatory responses and pyroptosis in lung epithelial BEAS-2B and A549 cells. (A-C)** BEAS-2B cells were transfected with pCMV3-SARS-CoV-2 S (pCMV-S), pTT5-SARS-CoV-2(M), and pTT5-SARS-CoV-2(N) for 24 h. (**A)** mRNA levels of inflammation-related genes were measured by qRT-PCR. (**B)** mRNA levels of inflammasome-related genes were measured by qRT-PCR. (**C)** Levels of IL-1β release in the supernatant were detected by ELISA. (**D)** Cell viability of BEAS-2B cells was measured using the MTT assay after transfection with pCMV-S (0.5, 1, 2, 4, 8 μg) for 24 h. (**E-F)** BEAS-2B cells were transfected with pCMV-S and pre-treated with cell death inhibitors (Fer-1, VX765, and Nec-1) or mock treatment. (**E)** Cell viability was measured using the MTT assay. (**F)** Levels of IL-1β release in the supernatant were detected by ELISA. (**G-L)** A549 cells were transfected with pCMV-S (0.5, 1, 2 μg) for 24 h. (**G)** WB analysis of SARS-CoV-2 S protein expression. (**H)** TEM observation of membrane rupture in cells. N, nucleus. Scale bar, 5 μm and 2 μm. (**I)** An IF staining assay showed the co-localization of Dio (green) and GSDMD (blue) using confocal microscopy (upper). Scale bars, 10 μm. Manders' overlap coefficient quantification for Dio and GSDMD co-localization in cells is presented in a bar graph (lower). (**J)** WB analysis of GSDMD and IL-1β protein expression. (**K)** LDH release assay (left) and ELISA (right) were used to measure plasma membrane rupture and IL-1β release levels in the supernatant, respectively. (**L)** WB analysis of NLRP3, NLRC4, and AIM2 proteins. (**M-O)** A549 cells were pre-treated with CY09 (20 μM, for 3 h) and followed by the transfection with pCMV-S (1 μg, for 24 h) or mock transfection. (**M)** WB analysis of NLRP3 inflammasome activation (NLRP3, ASC, and p10) and pyroptosis-related proteins (GSDMD and IL-1β). (**N)** Cell viability was measured using the MTT assay. (**O)** LDH release assay (left) and ELISA (right) were used to measure plasma membrane rupture and IL-1β release levels in the supernatant, respectively. Data are represented as mean ± SD. *, *P* < 0.05, compare to the control group or corresponding group.


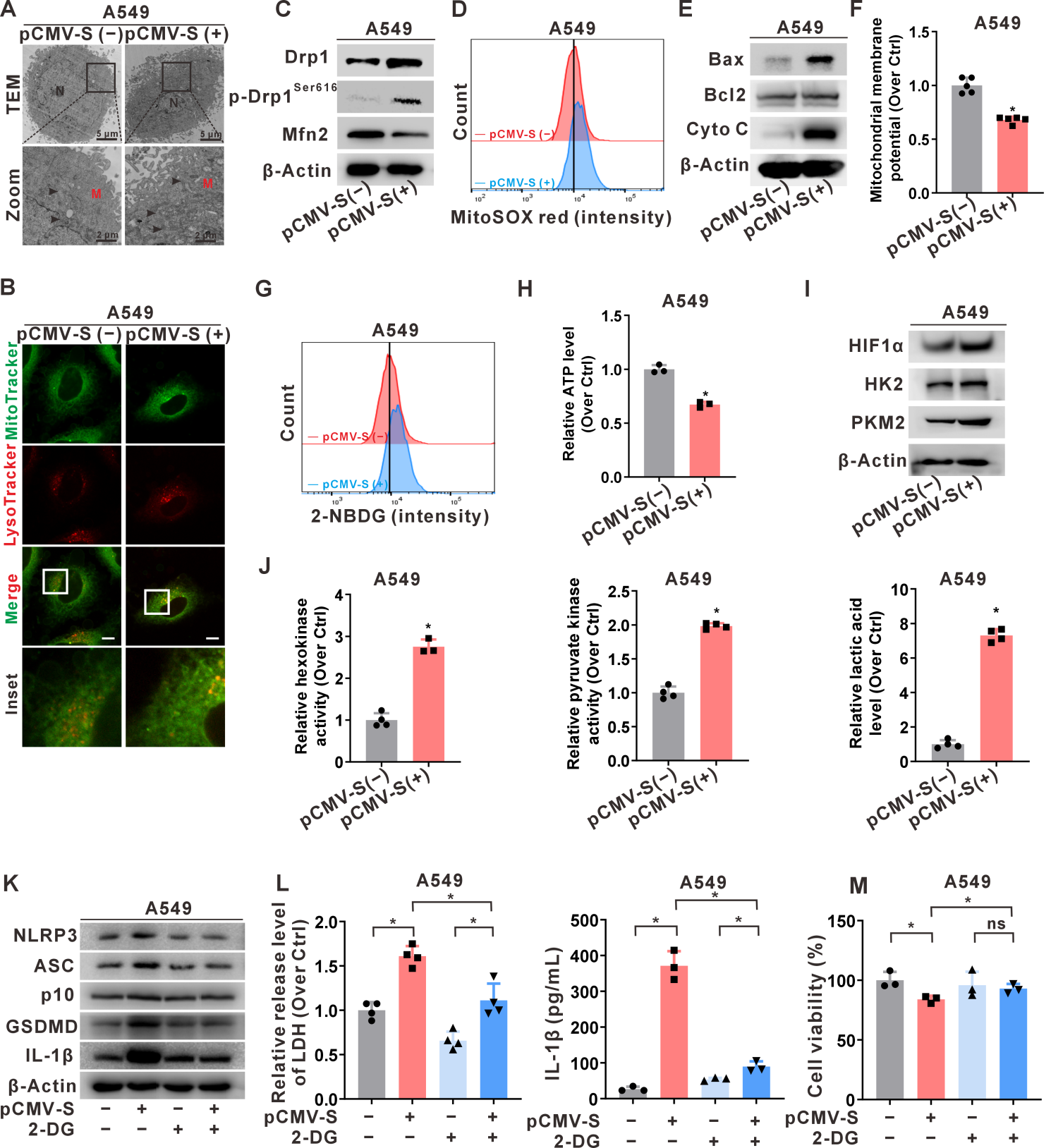


**Fig. S2. Regulation of SARS-CoV-2 S protein expression on mitochondrial quality control and metabolic reprogramming in lung epithelial A549 cells. (A-J)** A549 cells were transfected with pCMV3-SARS-CoV-2 S (pCMV-S, 1 μg for 24 h) to induce the S protein expression. (**A)** TEM observation of mitochondrial morphology in cells. N, nucleus. M, mitochondria. Scale bar, 5 μm and 2 μm. (**B)** IF staining of MitoTracker (green) and LysoTracker (red) was used to assess the intensity and co-localization in cells. (**C)** WB analysis of mitochondrial dynamics proteins (Drp1, p-Drp1^Ser616^, and Mfn2). (**D)** FCM assay of mitochondrial ROS levels using MitoSOX (red) in cells. (**E)** WB analysis of mitochondrial damage-related proteins (Bax, Bcl2, and Cyto C). (**F)** JC-1 assay of mitochondrial membrane potential. (**G)** FCM assay of glucose transport activity using fluorescent glucose analog 2-NBDG in cells. (**H)** Detection of ATP content in cells. (**I)** WB analysis of glycolysis-related proteins (HIF1α, HK2, and PKM2) levels. (**J)** Levels of glycolysis rate-limiting enzymes (hexokinase and pyruvate kinase) activities and glycolysis product lactic acid. (**K-M)** A549 cells were pre-treated with the hexokinase inhibitor 2-deoxy-D-glucose (2-DG, 20 μM for 3 h), followed by transfection with pCMV-S (1 μg for 24 h) or not. (**K)** WB analysis of NLRP3 inflammasome activation (NLRP3, ASC, and p10) and pyroptosis-related proteins (GSDMD and IL-1β). (**L)** LDH release assay and ELISA detection of IL-1β release levels in the supernatant. (**M)** Cell viability was measured using the MTT assay. Data are represented as mean ± SD. *, *P* < 0.05, compare to the control group or corresponding group.


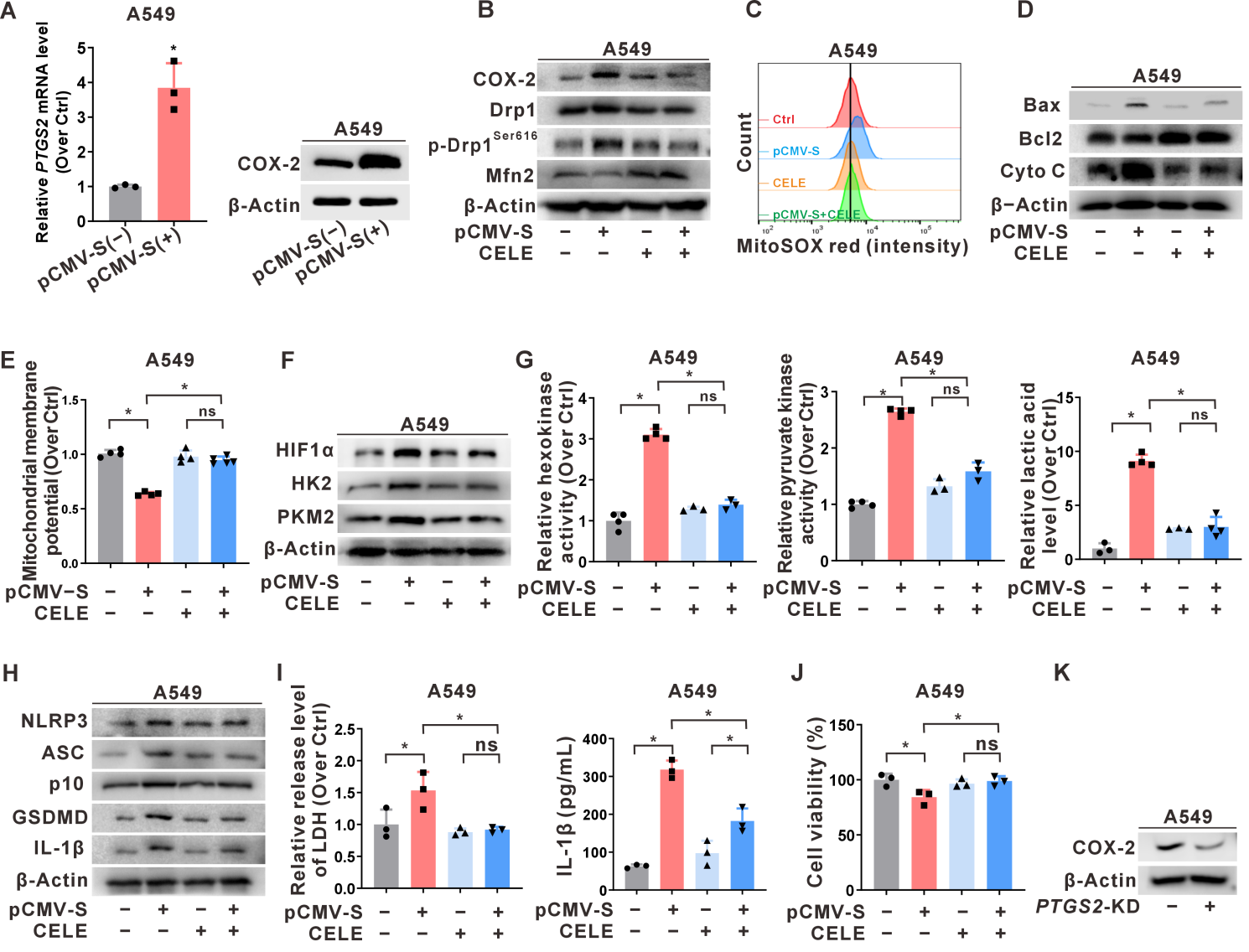


**Fig. S3. SARS-CoV-2 S protein-induced COX-2 regulates mitochondrial quality control and metabolic reprogramming, inhibiting pyroptosis in A549 cells. (A-J)** A549 cells were transfected with pCMV3-SARS-CoV-2 S (pCMV-S, 1 μg for 24 h) and pre-treated with CELE (40 μM for 3h) or not. (**A)** qRT-PCR analysis of *PTGS2* mRNA levels (left) and WB analysis of COX-2 protein levels (right). (**B)** WB analysis of COX-2 and mitochondrial dynamics proteins (Drp1, p-Drp1^Ser616^, and Mfn2). (**C)** FCM assay of mitochondrial ROS levels using MitoSOX (red) in cells. (**D)** WB analysis of mitochondrial damage-related proteins (Bax, Bcl2, and Cyto C). (**E)** JC-1 assay of mitochondrial membrane potential. (**F)** WB analysis of glycolysis-related proteins (HIF1α, HK2, and PKM2). (**G)** Levels of glycolysis rate-limiting enzymes (hexokinase and pyruvate kinase) activities and glycolysis product lactic acid. (**H)** WB analysis of NLRP3 inflammasome activation (NLRP3, ASC, and p10) and pyroptosis-related proteins (GSDMD and IL-1β). (**I)** LDH release assay and ELISA detection of IL-1β release levels in the supernatant. (**J)** Cell viability was measured using the MTT assay. (**K)** A stable COX-2 knockdown A549-*PTGS2*-KD cell was constructed using Cas9-*PTGS2* recombinant plasmid and COX-2 protein level was detected. Data are represented as mean ± SD. *, *P* < 0.05, compare to the control group or corresponding group.


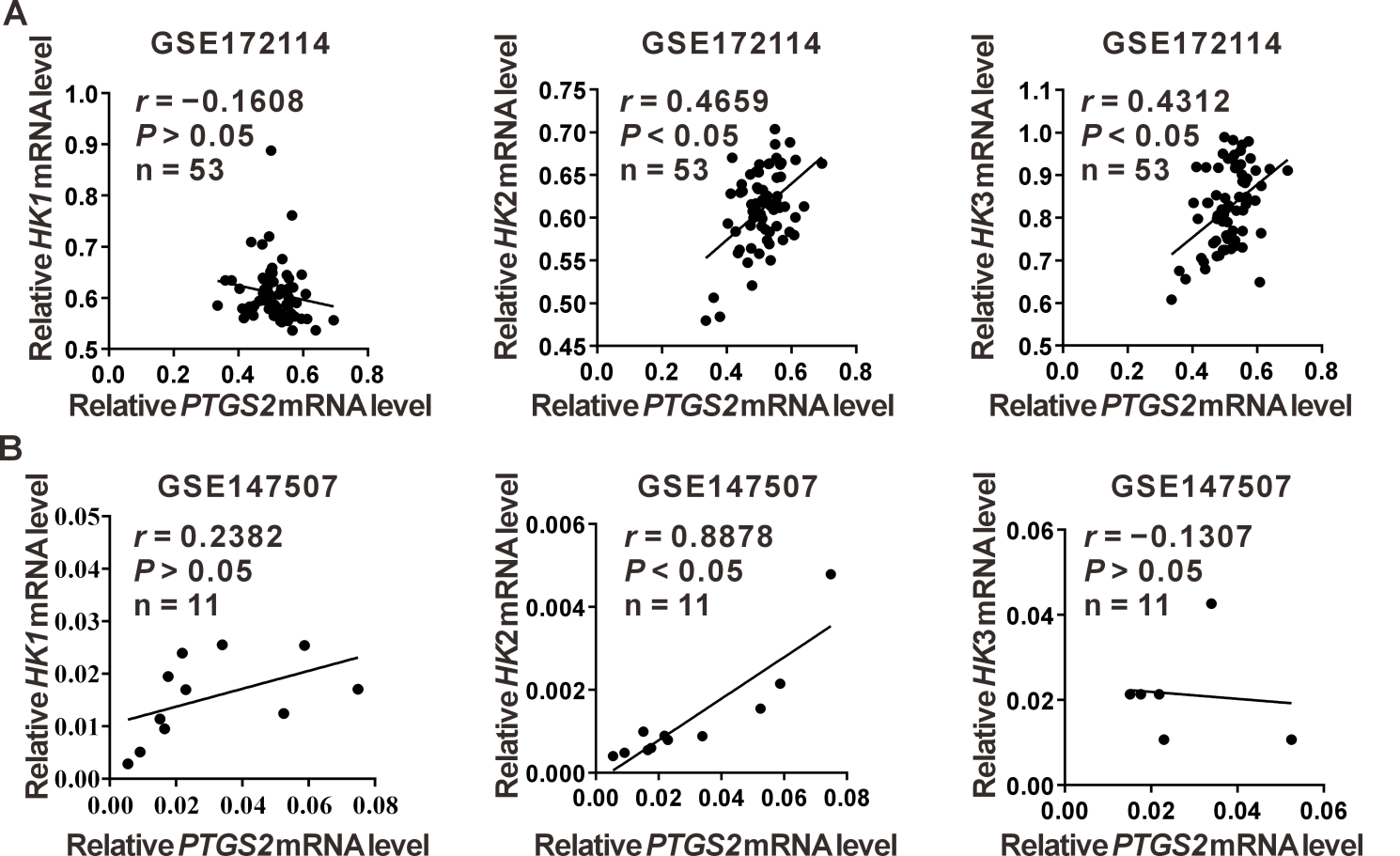


**Fig. S4. Correlation analysis between the relative mRNA levels of *PTGS2* and *HKs* in the GEO database.** The GEO database (GSE172114), retrieved from whole blood samples from patients with COVID-19 (n = 53) **(A)** and the GEO database (GSE147507), retrieved from SARS-CoV-2-infected A549 cell samples (n = 11) **(B)** were screened to assess the correlation between the relative mRNA level of *PTGS2* and *HKs*.


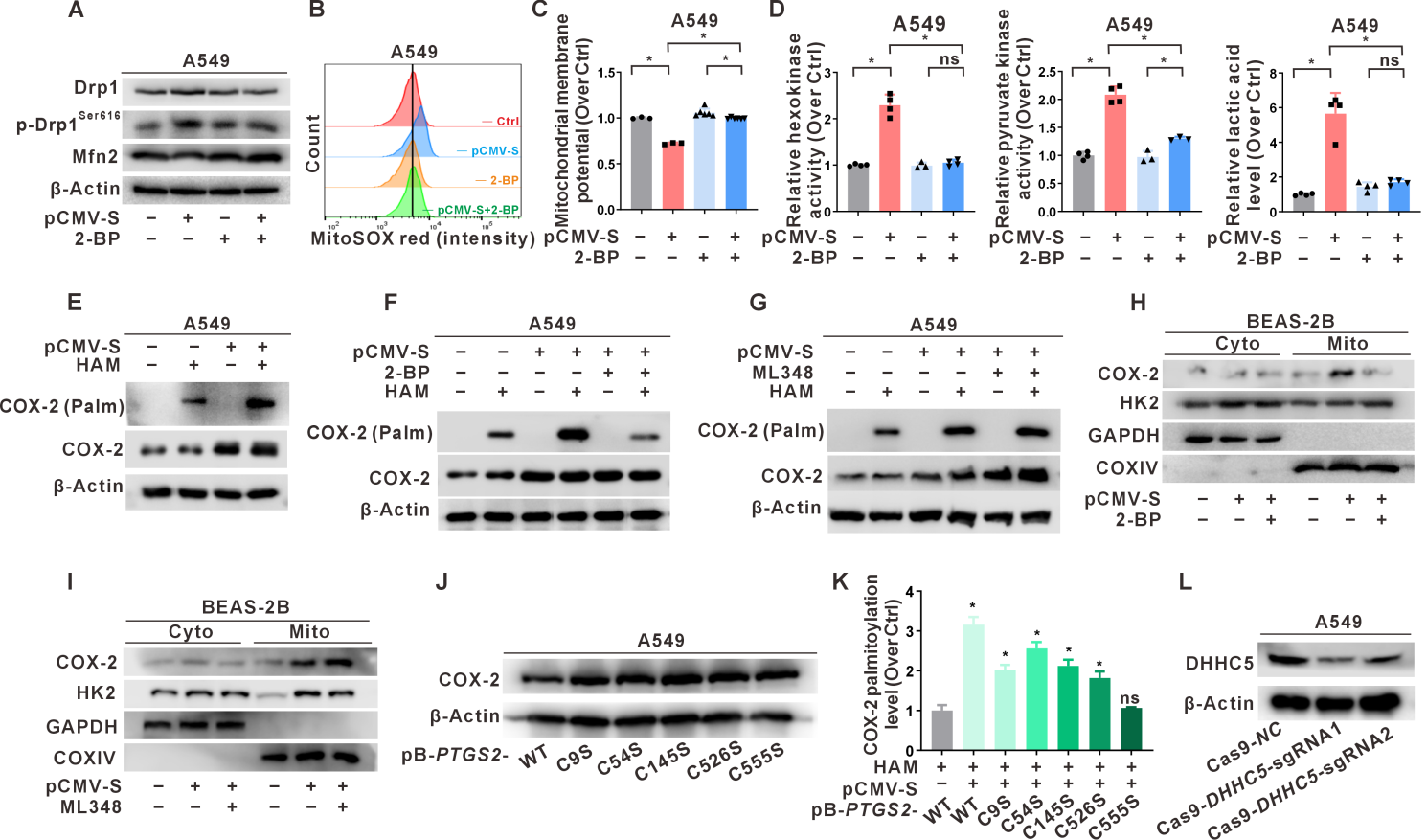


**Fig. S5. Regulation of mitochondrial COX-2^Cys555^ palmitoylation and its interaction with HK2 in SARS-CoV-2 S protein-expressing A549 cells. (A-D)** A549 cells were pre-treated with a non-specific palmitoylation inhibitor, 2-BP (40 μM, for 3 h), followed by transfection with pCMV-S (1 μg, for 24 h) or mock transfection. (**A)** WB analysis of mitochondrial dynamics proteins (Drp1, p-Drp1^Ser616^, and Mfn2). (**B)** FCM assay of mitochondrial ROS levels using MitoSOX (red) in cells. (**C)** JC-1 assay of mitochondrial membrane potential. (**D)** Levels of glycolysis rate-limiting enzymes (hexokinase and pyruvate kinase) activities and glycolysis product lactic acid. (**E-G)** The acyl-resin-assisted capture (Acyl-RAC) assay was performed to detect COX-2 palmitoylation of and to evaluate the effects of 2-BP and ML348 on this modification. (**H-I)** WB analysis of COX-2 and HK2 expression in cytoplasmic (Cyto) and mitochondrial (Mito) fractions. (**J)** WB detection of COX-2 level in A549 cells expressing wild-type (WT) or *S*-palmitoylation-deficient COX-2 variants (*PTGS2*-C5S, -C54S, -C145S, -C526S, and -C555S). (**K)** Quantitative analysis of COX-2 palmitoylation levels, as shown in Fig.5K. (**L)** A stable DHHC5 knockdown A549-*DHHC5*-KD cell was constructed using a Cas9-*DHHC5* recombinant plasmid, and the DHHC5 protein level was detected. Data are represented as mean ± SD. *, *P* < 0.05, compare to the control group or corresponding group.
